# Sustained benefit of compression therapy for preventing recurrent leg cellulitis: extended follow-up of a randomised controlled trial

**DOI:** 10.1016/j.eclinm.2026.104127

**Published:** 2026-08-04

**Authors:** Elizabeth Webb, Teresa Neeman, Francis J. Bowden, Jamie Gaida, Virginia Mumford, Bernie Bissett

**Affiliations:** a Clinical Hub for Interventional Research, John Curtin School of Medical Research, College of Science and Medicine, Australian National University, Canberra, Australia; b Physiotherapy Department, North Canberra Hospital, Canberra Health Services, Australia; c Biological Data Science Institute, College of Science and Medicine, Australian National University, Canberra, Australia; d Monash Rural Health, School of Rural Health, Faculty of Medicine, Nursing and Health Sciences, Monash University, Bendigo, Australia; e Discipline of Physiotherapy, Faculty of Health, University of Canberra, Canberra, Australia; f Australian Institute of Health Innovation, Macquarie University, Sydney, Australia

**Keywords:** Cellulitis, Recurrence, Oedema, Lymphoedema, Compression stockings

## Abstract

**Background:**

Recurrent cellulitis causes substantial morbidity, healthcare use, and repeated antibiotic exposure. Chronic oedema is a key modifiable risk factor for incident and recurrent cellulitis, yet preventive strategies targeting oedema remain underused. Our randomised controlled trial previously demonstrated that compression therapy reduced cellulitis recurrence, leading to early trial cessation for efficacy at interim analysis. We report extended follow-up outcomes.

**Methods:**

We conducted extended follow-up of a single-centre, open-label randomised controlled trial in an outpatient setting among Australian adults with chronic leg oedema and recurrent cellulitis. Participants were randomised 1:1 to receive cellulitis prevention education (control) or education plus compression therapy, stratified by prophylactic antibiotic use. After a pre-specified interim analysis demonstrated efficacy, recruitment ceased. Compression therapy was subsequently provided to control participants, with timing determined by participant preference and clinical availability. Follow-up continued until 18 months post-enrolment. The primary outcome was time to cellulitis recurrence. Secondary outcomes included cellulitis-related hospitalisation. Analyses were by intention-to-treat in the originally assigned groups. This study is registered with the Australian New Zealand Clinical Trials Registry (ACTRN12617000412336).

**Findings:**

Between June 2017 and March 2019, 183 individuals were screened and 90 were enrolled (45 per group). Median follow-up increased from 6 to 18 months. Cellulitis occurred in nine participants (20%) in the compression group and 21 (47%) in the control group (hazard ratio 0·23, 95% CI 0·10–0·52; p = 0·0001). Hospitalisation for recurrent cellulitis occurred in four (9%) compression participants and eight (18%) controls (hazard ratio 0·25, 95% CI 0·07–0·90). Adherence to compression therapy remained high throughout follow-up, with 85% of participants reporting garment use ≥4 days per week.

**Interpretation:**

Compression therapy provides a durable reduction in recurrent leg cellulitis and related hospitalisation in patients with chronic oedema. As an effective non-pharmacological intervention, compression therapy should be considered a first-line preventive strategy, with important implications for patient outcomes, antibiotic stewardship, and healthcare resource use.

**Funding:**

In-kind provision of compression garments by Haddenham Healthcare.

**Research in context**

**Evidence before this study**

We searched PubMed, Embase and Web of Science for studies published from database inception to June 1, 2017, using the search terms “cellulitis”, “erysipelas”, and “compression”, along with common synonyms. Reference lists of relevant articles were also screened. Only two articles were identified, both reporting before-and-after studies of Complete Decongestive Therapy (CDT), which combines compression therapy with skin care, exercise and manual lymphatic drainage, in patients with arm or leg lymphoedema. Arsenault and colleagues reported that, among 10 of 21 enrolled participants with follow-up data to 18 months, the cumulative annual incidence rate of hospitalisation for recurrent cellulitis reduced from 8·5 to 0·67 admissions per year. Ko and colleagues found that among 299 consecutively recruited participants, the incidence of cellulitis was reduced from 1·10 to 0·65 infections per patient per year.

Cellulitis prevention research has mainly focused on antibiotic prophylaxis, with few studies on modifying known risk factors such as chronic oedema. Consistent with our search findings, a 2017 Cochrane systematic review, *“Interventions for the prevention of recurrent erysipelas and cellulitis”*, included no randomised trials evaluating risk factor modification, despite their key role in cellulitis pathogenesis. Together, these findings demonstrate a substantial evidence gap and highlight the need for randomised trials evaluating chronic oedema management and other risk factor modification strategies for the prevention of recurrent cellulitis. The Cochrane review similarly recommended incorporating such strategies into cellulitis prevention programmes and called for trials evaluating their efficacy.

**Added value of this study**

To our knowledge, we conducted the first randomised controlled trial in people with recurrent cellulitis and chronic leg swelling to evaluate whether compression therapy combined with cellulitis prevention education, compared to education alone, reduced the risk of recurrent cellulitis. The trial was stopped early for efficacy after an interim analysis showed a marked reduction in recurrent cellulitis among participants receiving compression therapy. Extended follow-up now shows that the preventive effect of compression therapy is sustained and associated with fewer cellulitis-related hospital admissions. High adherence indicates compression therapy is both feasible and acceptable to patients.

**Implications of all the available evidence**

These findings support the management of chronic leg oedema with compression therapy as a core component of cellulitis prevention. Compression therapy is an effective, acceptable, non-pharmacological strategy with potential benefits for healthcare utilisation, antibiotic stewardship, and patient outcomes.

## Introduction

Cellulitis is a common bacterial infection of the skin and subcutaneous tissue with substantial global health impact.^1^^,^^2^ In 2019, an estimated 43 million cases of cellulitis occurred globally (555 per 100,000 population).^3^ The infection most frequently affects the lower limbs^4^, ^5^, ^6^ and accounts for a substantial proportion of both inpatient and outpatient presentations.^1^ Beyond the impact of individual episodes, its burden is amplified by a tendency to recur,^1^^,^^7^ leading to cumulative morbidity, repeated antibiotic exposure, and escalating healthcare utilisation.

Recurrence is common, with an estimated 26–47% of patients experiencing one or more subsequent episodes within a few years.^6^^,^^8^, ^9^, ^10^, ^11^ These recurrent infections are often more severe than initial episodes and are associated with longer hospital admissions and increased healthcare utilisation.^10^^,^^11^ Given that recurrent episodes account for approximately 50% of cellulitis-related hospitalisations,^9^^,^^11^ targeting recurrence represents a particularly high-value strategy for reducing overall morbidity and healthcare burden.

Chronic oedema, present in approximately 45–70% of patients with cellulitis,^12^^,^^13^ is one of the strongest risk factors for both incident and recurrent disease.^4^^,^^9^^,^^11^^,^^14^ Infection can cause or exacerbate chronic oedema, creating a self-perpetuating cycle that increases susceptibility to further episodes.^9^ More broadly, cellulitis frequently arises in the context of underlying risk factors, including oedema, obesity, fungal infections, and impaired skin integrity.^4^^,^^6^^,^^11^^,^^14^, ^15^, ^16^ Despite this, management is typically focused on treatment of the acute infection, with little emphasis on modifying predisposing factors for long-term prevention.

Antibiotic prophylaxis is a preventive strategy supported by clinical trial evidence; however, its benefit diminishes after cessation,^12^ and long-term use is associated with adverse effects and must be justified from an antibiotic stewardship perspective.^17^ Although many clinical guidelines recommend addressing modifiable risk factors before instituting antibiotic prophylaxis,^18^^,^^19^ implementation remains inconsistent, in part because high-quality evidence supporting such preventive interventions is limited.^20^

We previously conducted a randomised clinical trial to evaluate whether compression therapy could prevent recurrent leg cellulitis in patients with chronic oedema.^21^ An interim analysis demonstrated that compression therapy significantly reduced the risk of repeat infections by almost 80% (hazard ratio, 0·23, 95% CI 0·09–0·59).^21^ This finding met the trial's pre-specified stopping criteria for efficacy, prompting early cessation of recruitment and the randomisation phase of the trial, with compression therapy then offered to all participants originally assigned to the control group. Longitudinal follow-up of all participants continued for up to 18 months after enrolment. As a result, median follow-up was extended from 6 months (186 days) at the time of the interim analysis to 18 months (546 days) in the current analysis, allowing assessment of the durability of treatment effects.

This paper reports extended follow-up outcomes from the trial, using an intention-to-treat approach to examine the longer-term effects of compression therapy on cellulitis recurrence, cellulitis-related hospitalisation, quality of life, and leg volume.

## Methods

### Trial design and oversight

This single centre, open-label, randomised, phase III trial was conducted at North Canberra Hospital (previously Calvary Public Hospital Bruce), in Australia from June 2017 until October 2020. The protocol, methodology, and statistical analysis plan, including details of the interim analysis have previously been published.^21^^,^^22^ Briefly, participants were randomly allocated in a 1:1 ratio to receive education on cellulitis prevention, or the same education plus compression therapy, with randomisation stratified by prophylactic antibiotic use.

Following a pre-specified interim analysis and early stopping of the randomisation phase for efficacy, further recruitment was ceased and the planned follow-up duration was revised from three years to 18 months post enrolment for all participants. Participants originally assigned to the control group were subsequently offered compression therapy. The study is reported in accordance with the Consolidated Standards of Reporting Trials (CONSORT) 2010 statement.

### Ethics

The trial protocol was approved by three institutional Human Research Ethics Committees covering the authors’ affiliations and recruitment sites: Calvary Public Hospital Bruce (approval no. 53-2016), Australian Capital Territory Health (approval no. ETH.4·17·092), and the University of Canberra (cross-institutional approval). The trial was prospectively registered with the Australian New Zealand Clinical Trials Registry on 22 March 2017 (ACTRN12617000412336). The study was conducted in accordance with the protocol, Good Clinical Practice guidelines, and the Declaration of Helsinki. Written informed consent was provided by all participants.

### Participants

Eligible participants were aged 18 years or older with a history of two or more episodes of cellulitis in the same leg within the two years preceding trial referral, and had chronic oedema (oedema persisting ≥3 months) in one or both legs with recurrent cellulitis. Patients were excluded if they were already wearing effective compression garments five or more days per week, had a contraindication to compression, were receiving end-of-life care, had a clinically unstable condition, or had a chronic wound requiring specialist management. The full eligibility criteria are available in the protocol.

Participants were recruited from two primary public hospitals, or through referrals from general practitioners in the local region.

### Interventions

Cellulitis prevention education was provided at the initial visit and reinforced at follow-up visits in both groups. Education addressed the benefits of appropriate skin care, weight management, prevention of interdigital fungal infections, and engaging in regular exercise.

Participants assigned to the compression group were prescribed two sets of compression garments and instructed to wear them daily during waking hours. Compression therapy was prescribed and fitted by accredited lymphoedema therapists and individualised according to leg size and shape, oedema severity, skin condition, participant preference, and ability to independently apply and remove garments. Participants with bilateral leg oedema were prescribed compression therapy for both legs. Garments were either custom made or off-the-shelf (pre-determined sizes), with replacement recommended every 6–12 months; replacements were funded by participants or, if eligible, through government schemes.

Short courses of compression bandaging (typically 3–5 days) were provided when clinically indicated to reduce oedema prior to garment measurement and fitting. Participants received standardised education on garment use, safety, laundering, and application and removal.

Participants in the compression group received two free sets of compression garments at trial commencement. Participants in the control group received two free sets of compression garments following a recurrent cellulitis episode or after early termination of the randomisation phase, at the time of crossover to compression therapy, with timing determined by clinical availability and participant preference.

### Outcomes and assessments

All assessments were completed by unmasked accredited lymphoedema therapists in the hospital outpatient department. Baseline demographics, leg volume and quality of life were collected prior to randomisation.

The primary outcome was time to first episode of recurrent cellulitis occurring in a leg identified as having chronic oedema at baseline. Secondary outcomes were time to cellulitis-related hospital admission and changes in leg volume and quality of life, assessed at 6-monthly intervals.

Recurrent cellulitis episodes and associated hospitalisations were identified by participant self-report at the time of infection or at 6-monthly follow-up appointments, with verification using medical records when available. Cellulitis was diagnosed by physicians independent to the trial. Recurrence was documented only when it occurred in a leg identified as having chronic oedema at baseline. Leg volume was measured using an optoelectronic imaging device (perometer) between 5·3 cm and 40 cm height from the ground, minimising the potential for measurement bias. Quality of life was assessed using both the quality of life measure for limb lymphoedema (LYMQOL)^23^ tool and the EuroQol Five Dimension Scale (EQ-5D-3L), as previously described. Intervention adherence was determined by self-reported frequency of garment use (days worn/week), and adverse device effects were captured by participant report during scheduled follow-up assessments.

### Statistical analysis

The original randomisation procedures, sample size calculation and interim stopping rules have been reported previously.^21^ For the extended follow-up, analyses of the primary and secondary outcomes were conducted according to the intention-to-treat principle, with participants analysed according to their original group allocation.

Participants were censored at trial withdrawal (e.g. death), loss to follow-up, or end of follow-up. Participants assigned to the control group were additionally censored at crossover to compression therapy, defined as the date of initial compression garment fitting. Data collected after crossover were excluded from between-group comparative analyses and were used for descriptive analyses only. This approach differed from the original analysis plan by incorporating early trial cessation with crossover, while preserving the randomised comparison.

Time-to-event outcomes (time to first recurrent cellulitis episode and cellulitis-related hospital admission) were summarised using Kaplan–Meier methods, with between-group differences assessed using the log-rank test. Although prophylactic antibiotic use was a stratification variable, it was not included as an adjustment variable because only four participants were receiving prophylaxis, with two in each treatment group. A per-protocol sensitivity analysis of time to first recurrent cellulitis episode was conducted using the same methods, with non-adherent participants in the compression group who wore their compression garments fewer than four days per week excluded from the analysis. For the exploratory analyses, hazard ratios for prespecified cellulitis risk factors were estimated using separate Cox proportional hazards models. Each model included one covariate of interest and the randomised treatment group. Proportional hazards assumptions were assessed using standard diagnostic methods.

Analyses of the secondary outcomes of mean change in leg volume and quality of life outcomes were restricted to data collected up to 12 months post-enrolment; no formal imputation of missing data was undertaken. The high rate of infection resulted in treatment crossover for control group participants, with those who developed cellulitis no longer contributing to subsequent control group assessments. This reduced the availability of control group data at 12 months and introduced potential bias, rendering the groups non-comparable over time; therefore, no formal between-group comparisons were undertaken. For leg volume, an additional descriptive analysis was performed to examine volume change from the last appointment before crossover to the first appointment after crossover. All analyses were performed using R (version 3·6·0).^24^

### Role of the funding source

North Canberra Hospital funded clinician time and research administration support. Haddenham Healthcare provided two sets of compression garments per participant in-kind. The funders had no role in the trial design, data collection, data analysis or reporting, or the decision to submit the results for publication, and did not have access to the trial data.

## Results

### Participants

**Fig. 1:**
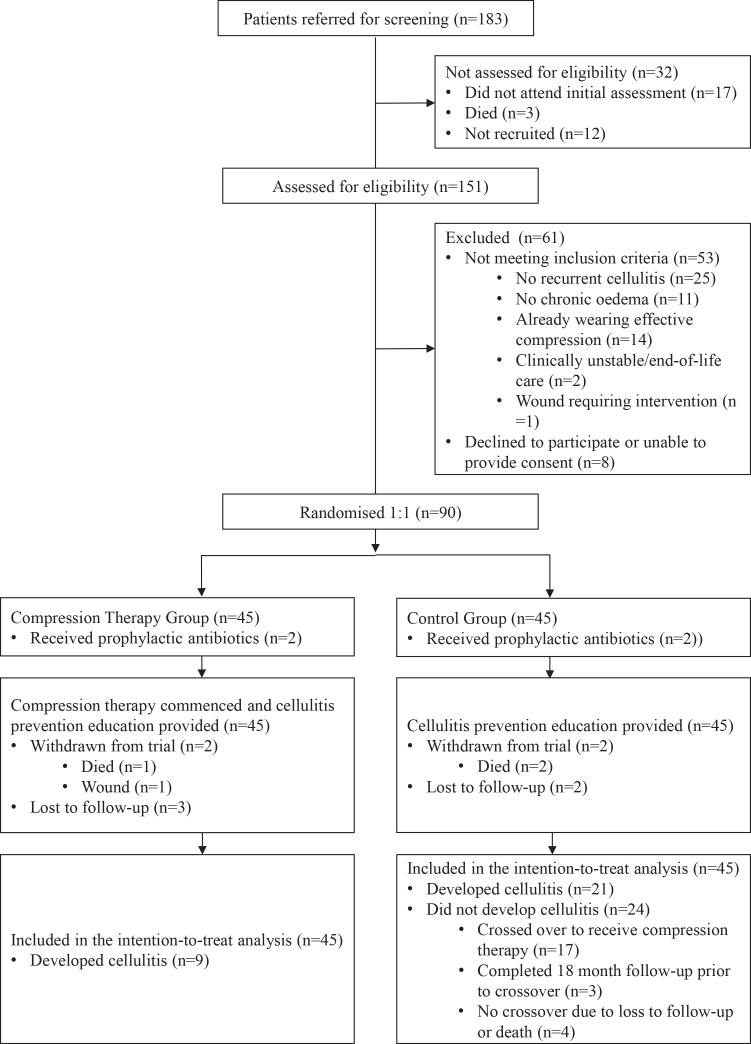
Trial profile.

Of 183 individuals screened, 90 participants were randomised, including 6 additional participants who were recruited whilst awaiting the interim analysis results; 45 to the compression group and 45 to the control group. Of the 93 individuals who were not enrolled, 32 did not proceed to a full eligibility assessment and 61 were excluded: 53 did not meet the eligibility criteria, and eight declined or were unable to provide consent (Fig. 1). Referrals originated from general practice in 21 cases (23%), inpatient settings in 16 (18%), the emergency department in 51 (57%), and self-referral from community advertising in 2 (2%). Randomisation was stopped early after the pre-specified interim analysis met stopping criteria for efficacy.

Over the duration of the trial, six participants (13%) in each group discontinued participation. In the compression group, three participants (7%) were withdrawn due to a wound (1), death (1), or relocating home (1), and a further three participants (7%) were lost to follow-up. Cellulitis recurred in one participant prior to withdrawal (Fig. 1). In the control group, four participants (9%) were withdrawn due to a wound (1), or death (3), and two participants (4%) were lost to follow-up. Cellulitis recurred in two participants prior to withdrawal (Fig. 1). Twenty participants without recurrent cellulitis were crossed over following interim analysis to receive compression therapy, however three completed the trial at the same time as crossover (Fig. 1). Of the participants who reported cellulitis recurrence during the trial, 77% (23/30) had their medical record checked or general practitioner contacted to verify diagnosis.

The median total follow-up time was 18 months (545 days in the compression group and 548 days in the control group). In the control group, median follow-up while remaining in the originally assigned group was 284 days. This shorter duration reflects the high proportion of participants who experienced recurrent cellulitis or who were event-free but were crossed over after interim analysis. Median follow-up after crossover to compression therapy was 190 days. Among control group participants who were not censored due to cellulitis, the median follow-up time until crossover or trial completion (whichever happened first) was 1 year (350 days).

Baseline demographics were similar between groups (Table 1). The majority of participants (78%) had bilateral chronic leg oedema, which had been present for 5 or more years for 61% of participants. The mean body mass index (BMI) was 41 kg/m^2^ (obese class III), with almost 75% of participants having a BMI of 35 kg/m^2^ or more (obese class II). Obesity was the most common factor identified by clinicians as contributing to chronic oedema, followed by surgery or trauma and venous hypertension. Race and ethnicity were not recorded in this study and therefore cannot be reported in the baseline characteristics.

**Table 1:** Baseline characteristics of the participants.

Characteristic	Compression (N = 45)	Control (N = 45)	Total (N = 90)
Age			
Mean^a^	65 ± 14·6	65 ± 13·0	65 ± 13·8
Median (IQR)	69 (52–75)	66 (58–73)	67 (55–74)
Female sex—no. (%)	21 (47)	23 (51)	44 (49)
Body-mass index^b^			
Mean^a^	40 ± 10·1	42 ± 9·6	41 ± 9·9
Median (IQR)	39 (31–47)	41 (34–47)	41 (34–47)
Chronic oedema in both legs—no. (%)	35 (78)	35 (78)	70 (78)
Duration of oedema—no. (%)			
1–5 yrs (%)	17 (38)	18 (40)	35 (39)
>5 yrs (%)	28 (62)	27 (60)	55 (61)
Episodes of cellulitis per leg in the 2 yr before trial referral^c^			
Mean^a^	2 ± 1·5	2 ± 1·4	2 ± 1·4
Median (IQR)	2 (1–2)	2 (0–2)	2 (0–2)
Hospital admission for cellulitis in the 2 yr before trial referral			
Mean^a^	1 ± 0·9	1 ± 1·5	1 ± 1·2
Median (IQR)	1 (0–2)	1 (0–1)	1 (0–2)
Leg volume—ml	4037 ± 1307	4291 ± 1300	4164 ± 1306
Quality of Life			
LYMQOL^d^			
Combined domain score; range 4–16^a^	8·3 ± 2·7	8·3 ± 2·4	8·3 ± 2·5
QoL assessment; range 0–10^a^	6 ± 1·7	6 ± 2·1	6 ± 1·9
EQ-5D-3L at 12 months (95% CI)^a^^,^^e^			
Visual analogue scale; range 0–100^a^	68 ± 15·6	62 ± 15·0	65 ± 15·4
Descriptive system; range 5–15^a^	7·7 ± 1·88	8·74 ± 2·47	8·2 ± 2·24
Prophylactic antibiotic use—no. (%)	2 (5)	2 (5)	4 (5)
Factors contributing to chronic oedema—no. (%)			
Obesity	29 (64)	29 (64)	58 (64)
Surgery/trauma	15 (33)	14 (31)	29 (32)
Venous Hypertension	16 (36)	12 (27)	28 (31)
Immobility	4 (9)	8 (18)	12 (13)
Primary Lymphoedema	3 (7)	2 (4)	5 (6)
Cancer	0 (0)	1 (2)	1 (1)
Other	7 (16)	3 (7)	10 (11)
Comorbidities—no. (%)			
Tinea pedis	14 (31)	17 (38)	31 (34)
Diabetes	10 (22)	14 (31)	24 (27)
Chronic Venous Insufficiency	13 (29)	12 (27)	25 (28)
Congestive Heart Failure	10 (22)	8 (18)	18 (20)

All participants were assumed to have some degree of oedema secondary to preceding cellulitis episodes.

a Values are reported as mean ± standard deviation.

b Body-mass index was calculated as weight (kg) divided by height squared (m^2^).

c Both legs were evaluated for prior cellulitis episodes.

d The quality-of-life measure for limb lymphoedema (LYMQOL) has two components that are assessed separately: a quality-of-life score (scores range from 0 to 10, with higher scores indicating better quality of life) and a combined score that encompasses four domains (symptoms, appearance, function, and mood), each scored at four levels (not at all, a little, quite a bit, or a lot; combined scores range from 4 to 16, with lower scores indicating better quality of life).

e The EuroQol Group 5–Dimensions 3-Level scale (EQ-5D-3L) has two components that are assessed separately: a visual analogue scale that assesses the overall health state (scores range from 0 [worst imaginable health state] to 100 [best imaginable health state]) and a descriptive system that assesses five dimensions of quality of life (mobility, personal care, usual activities, pain and discomfort, and anxiety and depression) at three levels (no problems, some problems, or extreme problems); total scores for the descriptive system range from 5 to 15, with lower scores indicating better quality of life.

### Intervention delivery and adherence

All participants in the compression group were prescribed two sets of compression garments, most commonly class II (23–32 mmHg) knee-high stockings. The time interval between the most recent cellulitis episode and trial enrolment varied across participants due to referral processes and clinic scheduling constraints but typically exceeded one to two months. The number of appointments required to deliver compression therapy varied according to individual participant needs. Adherence was high, with 85% (35/41) of participants reporting use on at least four days per week and 73% (30/41) reporting use on five or more days per week. One adverse device-related event was reported (an acute wound related to use of an application aid).

### Efficacy

#### Primary outcome: recurrent cellulitis

**Fig. 2:**
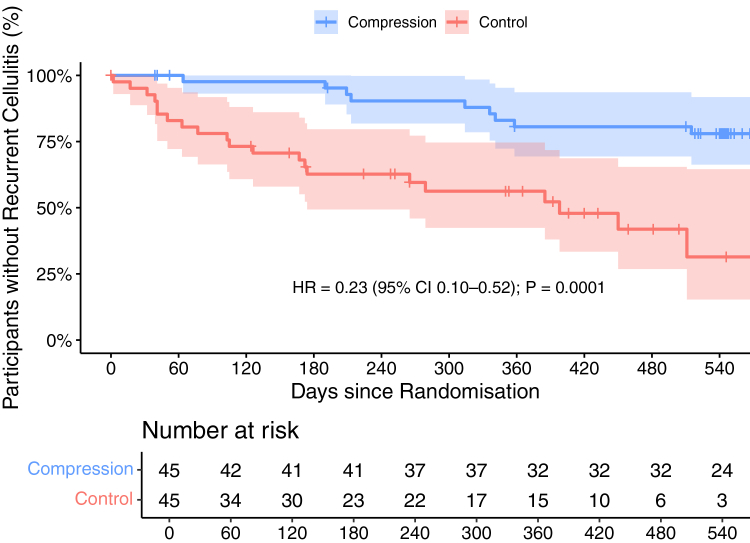
Freedom from recurrence of cellulitis over time. Kaplan–Meier curves depict freedom from recurrent cellulitis in the compression and control groups. Shaded bands represent 95% confidence intervals. The median duration of follow-up was 18 months (546 days). Censoring occurred when participants were withdrawn, lost to follow-up or crossover (control group only). Within the control group, three participants finished the trial before crossover.

Recurrent cellulitis occurred in nine participants (20%) in the compression group and 21 participants (47%) in the control group. Receiving compression therapy was associated with a 77% reduction in risk of recurrence (hazard ratio 0·23, 95% CI 0·10–0·52; p = 0·0001) (Fig. 2 and Table 2). This was a 27% absolute difference in event rates, giving a number needed to treat of 4 (95% CI, 3–13).

**Table 2:** Time to event outcomes.

Outcome	Compression	Control	Absolute risk difference, % points^a^	Effect estimate (95% CI)
Recurrent cellulitis, n (%)	9/45 (20)	21/45 (47)	−27	HR 0·23 (0·10–0·52); p = 0·0001 RR 0·32 (0·16–0·62)
Recurrent cellulitis, per-protocol sensitivity analysis^b^, n (%)	6/39 (15)	21/45 (47)	−32	HR 0·17 (0·07–0·43) RR 0·25 (0·11–0·56)
Hospitalisation for recurrent cellulitis, n (%)	4/45 (9)	8/45 (18)	−9	HR 0·25 (0·07–0·90)

Hazard ratios were estimated using unadjusted Cox proportional hazards models including trial group only.

Relative risks were calculated as the ratio of Kaplan–Meier estimated cumulative incidences at 18 months.

a Absolute risk difference is the simple difference in observed proportions (compression minus control).

b Per-protocol sensitivity analysis excluding participants who did not adhere to compression therapy. Six participants in the intervention group were excluded because compression garments were worn on fewer than four days per week.

For the per-protocol sensitivity analysis, six compression group participants in the compression group were excluded because they reported wearing their garments fewer than four days per week. Recurrent cellulitis occurred in six of 39 compression group participants (15%) and 21 of 45 control group participants (47%), corresponding to an 83% reduction in the risk of recurrence with compression therapy (hazard ratio 0·17, 95% CI 0·07–0·43) (Table 2).

Exploratory analyses identified a higher risk of recurrence among participants with three or more cellulitis episodes per leg in the two years before enrolment (hazard ratio 2·44, 95% CI 1·05–5·66). No significant associations were observed for baseline BMI ≥40 kg/m^2^, or the development of tinea or toe-web intertrigo, or wounds during follow-up (Table S1 in the Supplementary Appendix). The very high proportion of participants with obesity (median BMI 41, IQR 34–47) meant that few participants of normal weight were available for comparison, which may have limited the ability to detect differences between BMI categories.

#### Secondary outcomes

##### Hospitalisation

**Fig. 3:**
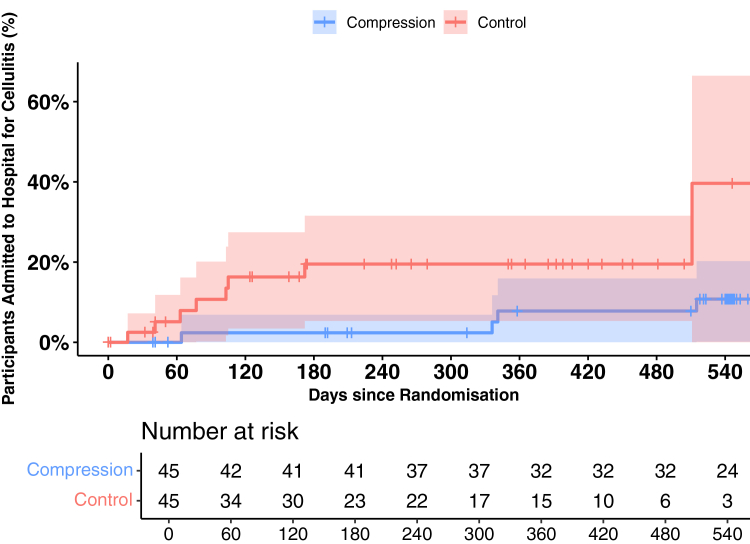
Participants admitted to hospital for cellulitis recurrence over time. Kaplan–Meier curves depict the cumulative incidence of hospital admission for recurrent cellulitis in the compression and control groups. Shaded bands represent 95% confidence intervals. The median duration of follow-up was 18 months (546 days). Censoring occurred when participants were withdrawn, lost to follow-up, had an episode of cellulitis without hospitalisation, or crossed over (control group only). Within the control group, three participants finished the trial before crossover.

Hospitalisation for cellulitis occurred in four participants (9%) in the compression group and eight participants (18%) in the control group. Compression therapy was associated with a 75% reduction in risk of hospitalisation (hazard ratio 0·25, 95% CI 0·07–0·90) (Fig. 3 and Table 2). Admissions occurred earlier and more frequently in the control group.

##### Leg volume

At 12 months, mean leg volume (measured between 5·3 cm and 40 cm height from the ground) reduced in the compression group by 151 mL (95% CI −219 to −81) but showed no meaningful change in the control group (58 mL; 95% CI −21 to 365). Control group participants’ mean leg volume reduced by 110 mL (95% CI −196 to −24) following crossover (Table 3).

**Table 3:** Descriptive analysis of secondary outcomes.

Outcome	Compression mean change (95% CI)	Control mean change (95% CI)
Change in leg volume at 12 months (95% CI)^a^		
Change in volume—ml	−150 (−219 to −81)	58 (−21 to 365)
Change in Leg volume from pre to post crossover (95% CI)		
Change in volume—ml		−110 (−196 to −24)
Change in LYMQOL at 12 months (95% CI)^a^^,^^b^		
Combined domain score; range 4–16	−1·48 (−2·19 to −0·77)	−0·88 (−1·76 to 0·00)
QoL assessment; range 0–10	0·59 (0·05 to 1·12)	−0·06 (−0·74 to 0·63)
Change in EQ-5D-3L at 12 months (95% CI)^a^^,^^c^		
Visual analogue scale; range 0–100	−2·53 (−9·42 to 4·38)	−4·13 (−16·28 to 8·03)
Descriptive system; range 5–15	−0·64 (−1·79 to 0·51)	−0·39 (−1·88 to 1·10)

a Analysis only included data from participants in their originally assigned groups. Data from the control group following crossover was excluded. At 12 months, data were available for 39 compression group participants, and 19 control group participants who remained in their originally assigned group. Outcomes show mean change from baseline.

b The quality-of-life measure for limb lymphoedema (LYMQOL) has two components that are assessed separately: a quality-of-life score (scores range from 0 to 10, with higher scores indicating better quality of life) and a combined score that encompasses four domains (symptoms, appearance, function, and mood), each scored at four levels (not at all, a little, quite a bit, or a lot; combined scores range from 4 to 16, with lower scores indicating better quality of life).

c The EuroQol Group 5–Dimensions 3-Level scale (EQ-5D-3L) has two components that are assessed separately: a visual analogue scale that assesses the overall health state (scores range from 0 [worst imaginable health state] to 100 [best imaginable health state]) and a descriptive system that assesses five dimensions of quality of life (mobility, personal care, usual activities, pain and discomfort, and anxiety and depression) at three levels (no problems, some problems, or extreme problems); total scores for the descriptive system range from 5 to 15, with lower scores indicating better quality of life.

##### Quality of life

*LYMQOL and EQ-5D-3L:* At 12 months, the compression group demonstrated improvement in the LYMQOL combined domain score (mean change −1·48; 95% CI −2·19 to −0·77) and in the generic quality of life assessment (mean change 0·59; 95% CI 0·05–1·12), but showed no change in the EQ-5D scores. No within-group changes in the LYMQOL or EQ-5D scores were demonstrated in the control group.

The ED-5D and LYMQOL results are shown in Table 3.

## Discussion

In this extended follow-up of a randomised clinical trial, the substantial reduction in cellulitis recurrence associated with compression therapy was maintained beyond the interim analysis period. With median follow-up increasing from 6 to 18 months, participants receiving compression therapy continued to experience markedly lower rates of recurrent cellulitis (20% versus 47%) and hospitalisation for recurrence (9% versus 18%), with the previously non-significant trend toward reduced hospitalisation reaching statistical significance. Reductions in recurrence and cellulitis-related hospitalisation indicate that compression therapy can prevent more severe infections, which is likely to reduce morbidity and healthcare utilisation.

Prevention of recurrent episodes reduces cumulative antibiotic exposure, advancing antibiotic stewardship, particularly given that cellulitis and other soft-tissue infections account for approximately 18% of outpatient antibiotic prescriptions.^25^ Although antibiotic prophylaxis is the only other commonly recommended, evidence-based intervention for preventing recurrent cellulitis,^20^^,^^26^ its protective effect diminishes after cessation, and its benefit is reduced in high-risk groups, including patients with chronic oedema, obesity, or multiple prior episodes.^12^ Consistent with guideline recommendations to address modifiable risk factors before resorting to prophylactic antibiotics,^18^^,^^19^ our findings demonstrate that compression therapy provides an effective and sustainable strategy to reduce recurrence and should be considered a first-line preventive approach for patients with chronic oedema.

Our findings are consistent with recent research. A large cross-sectional study across 40 sites in nine countries (n = 7477) found that 37% had experienced cellulitis, with 16% reporting an episode in the preceding year.^27^ Cellulitis risk increased with oedema severity (International Society of Lymphology stage II, odds ratio [OR] 2·04; stage III, OR 4·88), whereas controlled swelling reduced risk (OR 0·59). A recent randomised trial (n = 73) reported a comparable reduction in recurrence with compression therapy versus no compression (14% versus 38%; absolute reduction 24%).^28^ However, limitations in reporting and study design constrain interpretation. Unlike the present study, chronic leg oedema was not required for eligibility, which may account for the somewhat lower overall recurrence rates observed, given oedema's established role as a key risk factor.

Adherence to compression therapy remained high over the extended follow-up period, with 85% of participants wearing their garments on four or more days per week, supporting the acceptability and feasibility of sustained use. The per-protocol sensitivity analysis, which excluded non-adherent participants, demonstrated a stronger treatment effect, further supporting the importance of regular compression therapy use. Despite this, access to compression therapy remains challenging in many settings, reflecting health practitioners’ limited understanding and underdiagnosis of chronic oedema, as well as insufficient awareness of the benefits of compression therapy. Upfront costs and limited access to appropriately trained clinicians are additional barriers, particularly in low- and middle-income countries. Furthermore, poorly prescribed or ill-fitting garments can undermine adherence and contribute to clinician reluctance to recommend this treatment. Improved awareness among health professionals and the public regarding chronic oedema and its management, together with better resourcing, including garment funding and clinician training, are required to support broader and more effective provision of compression therapy.

The economic case for compression therapy is supported by a within-trial cost analysis demonstrating that, despite higher upfront costs, compression therapy was cost-saving from a health system perspective through prevention of recurrent cellulitis, largely due to avoided hospitalisations.^29^ The sustained reduction in recurrence-related hospitalisation over extended follow-up further supports compression therapy as a resource-efficient intervention, although cost-effectiveness will vary across healthcare systems.

Several considerations should be taken into account when interpreting and applying these findings. First, there is no gold standard diagnosis for cellulitis, and misdiagnosis is common.^30^ Additionally, some conditions misdiagnosed as cellulitis, particularly those resulting from venous disease, such as stasis dermatitis,^30^ may also improve with compression therapy. Consequently, some of the observed treatment effect may have resulted from reductions in episodes of these conditions rather than exclusively from reductions in cellulitis recurrence. However, this pragmatic trial approach reflects standard clinical practice, where diagnostic uncertainty is common. Whether the observed benefit arose exclusively from a true reduction in cellulitis recurrence or partly from improved management of venous disease, the intervention is likely to provide meaningful benefits to patients while reducing antibiotic use.

Second, the findings may have limited generalisability to patients with cellulitis in the absence of oedema or at other anatomical sites, or to populations with substantially different risk profiles. Because race and ethnicity data were not collected, we could not assess whether findings differed across racial or ethnic groups. The study cohort was characterised by chronic leg oedema, recurrent cellulitis and a high prevalence of obesity, reflecting a population with particularly high risk of recurrence.^6^^,^^9^^,^^10^^,^^27^ Although the proposed mechanism of action of compression therapy relates primarily to oedema control, including improvements in local immune function and skin integrity,^21^ and is therefore unlikely to be restricted to patients with recurrent infections and obesity, the magnitude of benefit observed in this trial is likely to be smaller in lower-risk populations.

Finally, appropriate prescription of compression therapy requires clinical expertise, which may limit translation and scalability in some settings. Therapist blinding was not possible due to the nature of the intervention. Cellulitis episodes, intervention adherence, and adverse device effects were at least partly ascertained through participant report, which may have introduced self-reporting bias, outcome misclassification, and uncertainty in event timing, although these limitations likely affected both groups similarly. Although loss to follow-up could not be definitively assumed to be random, attrition was balanced between groups (13% in each arm), and there were no apparent baseline differences between completers and non-completers, making substantial attrition bias unlikely. In addition, provision of compression therapy to control participants after the interim analysis limited formal comparative assessment beyond that time point.

This follow-up study extends our original findings by demonstrating that compression therapy provides a sustained reduction in recurrent cellulitis and recurrence-related hospitalisation among patients with chronic oedema. These findings support compression therapy as an effective, non-pharmacological strategy for cellulitis prevention, with implications for patient outcomes, antibiotic stewardship, and healthcare resource use. Future research should focus on replication in populations with lower risk profiles, implementation strategies, integration into clinical pathways, and evaluation of longer-term patient-centred outcomes.

## Contributors

EW contributed to conceptualisation, methodology, investigation, formal analysis, data curation, funding acquisition, validation, and writing—original draft; accessed and verified the data; and approved the final manuscript. BB contributed to conceptualisation, methodology, investigation, and resources. TN contributed to conceptualisation, methodology, and formal analysis, and accessed and verified data. VM contributed to methodology and formal analysis (economic analysis). BB, TN, FB, VM, and JG contributed to supervision, methodology refinement, writing—review and editing, and approved the final manuscript.

## Data sharing statement

Individual participant data collected for this study will not be made available because ethical approval for data sharing was not obtained from participants at the time of consent.

## Declaration of interests

EW reports travel support from conference and meeting organisers (the International Lymphoedema Framework, the Australasian Lymphology Association, Wounds Australia, and the Australian Physiotherapy Association) to present at scientific meetings and has delivered educational webinars for several organisations and companies without receiving personal honoraria. EW served as an unpaid committee representative for the Australasian Lymphology Association and the Australian Physiotherapy Association.

## Appendix A Supplementary data

Supplementary Appendix

Protocol and Statistical Analysis Plan
