# Supplementary material for: Sustained benefit of compression therapy for preventing recurrent leg cellulitis: extended follow-up of a randomised controlled trial
Source: eClinicalMedicine. 2026 Aug 4;98:104127. doi: 10.1016/j.eclinm.2026.104127 (PMC13469820; doi:10.1016/j.eclinm.2026.104127)
Supplement: Supplementary file 1 [file mmc1.pdf]

Supplementary Appendix

Sustained Benefit of Compression Therapy for Preventing Recurrent Leg Cellulitis: Extended Follow-up of a Randomised Controlled Trial

Elizabeth Webb, et al.

Table of contents

Table S1: Exploratory Analysis of Covariates known to Contribute Risk to Cellulitis Recurrence 2

Table S1: Exploratory Analysis of Covariates known to Contribute Risk to Cellulitis Recurrence

Covariate	Hazard ratio (95% CI)
Baseline body-mass index ≥40*	1·02 (0·48 – 2·15)
Episodes of cellulitis in the 2 years before trial referral: ≥3	2·44 (1·05 – 5·66)
Tinea or toe-web intertrigo reported or identified during the trial	1·72 (0·82 – 3·61)
Wound reported or identified during the trial†	1·75 (0·80 – 3·81)

*Body-mass index was calculated as weight (kg) divided by height squared (m^2).

† Excludes wounds resulting from cellulitis.

Hazard ratios were estimated using separate univariable Cox proportional hazards models. Each model included the randomised treatment group and one prespecified covariate (baseline BMI, ≥3 episodes of cellulitis in the 2 years before trial referral, tinea or toe-web intertrigo during the trial, or wound during the trial).
