# Supplementary material for: Sustained benefit of compression therapy for preventing recurrent leg cellulitis: extended follow-up of a randomised controlled trial
Source: eClinicalMedicine. 2026 Aug 4;98:104127. doi: 10.1016/j.eclinm.2026.104127 (PMC13469820; doi:10.1016/j.eclinm.2026.104127)
Supplement: Supplementary file 2 [file mmc2.pdf]

## This supplement contains the following items:

1. Original protocol, final protocol, summary of changes.

2. Original statistical analysis plan, final statistical analysis plan, summary of changes.

# Original Protocol

## Impact of Compression Therapy on Cellulitis (ICTOC)

### Introduction

The aim of this research is to investigate the functional impact of compression therapy (to manage chronic oedema) for adults who have chronic oedema and a history of recurrent cellulitis in a lower limb(s) who access health services within the ACT. Reducing cellulitis recurrence could decrease the burden on the healthcare system and may positively influence the quality of life (QOL) of individuals experiencing recurrent episodes of cellulitis. Whilst this study will be performed in adults receiving healthcare services in the ACT, it is hoped the results may provide insight for cellulitis management within Australia.

For definitions of cellulitis, chronic oedema and compression therapy, see appendix 1.

### Research Aim

This research will investigate the functional impact of compression therapy for adults accessing health services within the ACT who have chronic oedema and a history of recurrent cellulitis in a lower limb or limbs.

### Research Objectives

For adults with a history of chronic oedema and recurrent cellulitis who are accessing healthcare services within the ACT, we plan to investigate:

- If use of compression therapy delays the incidence of recurrent cellulitis in the affected limb/s (primary objective),
- If use of compression therapy decreases affected limb volume,
- If use of compression therapy improves quality of life, and
- If use of compression therapy delays incidence of hospitalisation for cellulitis in the affected limb/s.

### Description of the literature

Leg oedema is a potent risk factor for lower limb cellulitis (Cox, 2006). Lymphoedema, a type of chronic oedema, has been demonstrated to be the strongest risk factor for cellulitis, being a risk factor not only to an initial episode, but also for recurrence (Cox, 2006; Chlebicki & Oh, 2014; Dupuy et al., 1999; Inghammar, Rasmussen, & Linder, 2014). It is broadly accepted and understood that the relationship between cellulitis and oedema is a vicious cycle. Peripheral oedema predisposes

individuals to cellulitis and with each episode of cellulitis, the lymphatic system and hence lymphatic flow are further impaired, increasing residual oedema and heightening the risk of future cellulitis infections (Chlebicki & Oh, 2014). There is general consensus in the literature that, in addition to an antibiotic prescription, compression to manage oedema should be an adjuvant treatment for patients with chronic oedema who are experiencing cellulitis recurrence (Chlebicki & Oh, 2014; Cox, Colver, & Paterson, 1998; Morton & Swartz, 2004). Despite this common recommendation and the strong evidence supporting the relationship between oedema and cellulitis, there is a paucity of quality evidence to support the use of compression to manage chronic oedema to minimise or prevent cellulitis recurrence and therefore it is not yet standard practice.

**Method**

A randomised controlled trial (RCT) with cross-over will be used to assess the impact of compression therapy on clinical outcomes (time to next episode of cellulitis, QOL and oedema volume). Patients who meet the eligibility criteria (appendix 2) will be entered into the study and randomised to the intervention or control group using sealed envelopes. For patients in the control group, once an episode of cellulitis has occurred, they will cross-over into the intervention group to receive compression therapy. As prophylactic antibiotics may strongly influence recurrence of cellulitis (Morton & Swartz, 2004), randomisation of participants will be stratified by prophylactic antibiotic use. Appendix 3 shows the proposed participant allocation process.

Participants in the control group will receive education regarding the benefits of skin care, exercise, healthy weight management and prevention of tinea/fungal infections between toes, at an initial appointment. The intervention group will be provided with the same information but the initial appointment will also be used to plan appropriate compression therapy which will then be provided in subsequent additional appointments. The number of appointments necessary for provision of compression therapy will vary based on patient requirements. Following this, participants in both groups will be followed for up to three years at regular intervals (6-monthly) to have outcomes measures taken and to continue to receive the allocated treatment (education with or without compression therapy).

Level one lymphoedema trained therapists will provide the intervention and measure clinical outcomes and will not be blinded to patient group allocation. As the intervention and outcome measures will be performed during the same appointment by one therapist, it is logistically too difficult and labor intensive to blind therapists. Diagnosis of cellulitis will occur independently to the study and will be performed by doctors who are not involved in the trial and may or may not be aware of the patient's involvement in the study.  Patients will not be blinded due to ethical reasons and the visible nature of the treatment.

**Participant recruitment and enrolment**

Participants who meet the eligibility criteria (appendix 2) will be recruited from Calvary Public Hospital Bruce (CPHB), Canberra Hospital (CH) and general practitioners. Education and referral forms will be provided to recruitment sites to promote referrals. Participants from these sites must consent to referral to our service for the study, but do not need to consent to being a part of the study at time of referral.

Once referred to the service, an initial appointment with the CPHB Lymphoedema service will be organised. At this appointment, patients will receive a study information sheet, be informed about the study and be given the opportunity to consent or decline to participate. For those patients who have consented to participate, baseline measures will be completed prior to stratified randomisation.

To promote participation in the study, two free sets of compression garments will be offered. Compression garments are expensive and provide a barrier to treatment compliance. Participants in the intervention group will receive garments at intervention commencement; participants in the control group will receive them following their first recurrent cellulitis episode or upon study completion (for those who do not experience recurrence). In addition, at each appointment a therapist will inform each patient of changes in their limb oedema volume, which gives patients tangible feedback to support their attendance of the service.

From analysing ‘ACT patient administration system’ reports for CH and CPHB, we expect to receive between 50-150 referrals per year. As chronic oedema is associated with obesity, older age, cancer treatment and most likely poor mobility (Moffatt et al., 2004; Boza et al., 2011; Graf et al., 2013; Solaro et al., 2006), it is expected that a high proportion of study population will have comorbid conditions that may make attendance of appointments challenging. Therefore it is likely that a proportion of our referred patients will choose not to participate in the study. However clinical experience indicates once patients are attending our service, ongoing follow up rates are reasonably high.

**Sample size and duration of follow up**

Based on the assumptions that compression therapy will reduce incidence of recurrent cellulitis by 50% (Arsenault, Rielly, & Wise, 2011; Ko, Lerner, Klose, & Cosimi, 1998), and that the cellulitis recurrence rate is approximately 40% (Cox, 2006; Jorup-Ronstrom & Britton, 1987), in order to achieve a statistically significant effect (P=0.05) we plan to admit 152 participants (76 per arm) into the study for a follow up of three years or until 45 events of recurrent cellulitis occur. This sample size has been calculated for the primary objective.

## Selection bias

Use of randomisation will minimise selection bias and confounding. Stratification will ensure use of prophylactic antibiotics is not confounded with treatment assignment. Presence and distribution of other known potential confounding factors will be measured and reported. Intention to treat analysis will be used to prevent selection bias that may occur through loss to follow up of patients.

## Internal Validity

Use of an RCT and use of validated measurement tools support the internal validity of this research. The lack of blinding of therapists and patients has the potential to induce surveillance and recall bias and lead to differential measurement error in the reporting of cellulitis recurrence. To minimise the possibility of this, where possible accuracy of self-reporting of recurrence will be checked with the patient's GP or medical record (from Calvary Public Hospital Bruce and Canberra Hospital and Health Services). Diagnosis of cellulitis by doctors external to the study and use of perometry (appendix 4) to measure limb volume will minimise measurement bias and thus differential measurement error. Maintenance of the perometer will be performed to prevent non-differential measurement error that could result from machine error.

Patients in the intervention group will attend more appointments than the control group. This systematic difference in clinician contact could influence patient's perceived benefit, allowing potential bias in self-reported measures (LYMQOL).

## Generalisability

The target population is Australian adults who have chronic leg oedema and experience recurrent cellulitis. This study sample will be somewhat representative of this population, however as the sample will likely be largely from public hospitals within the ACT and must meet the selection criteria of having two or more episodes of cellulitis in the past 2 years, they may have more severe disease progression and be of differing health and socioeconomic status to the target population.

The patient demographics will be recorded and reported with the final results so that the characteristics of the study population are clear and able to compared and contrasted to other populations of interest.

## Data Collection

Outcome measures will be collected at predetermined times using a set of tools. Patient demographics and identified potential confounders will be measured.

Table 1 lists the measurement tools, the method and time frame for use of each tool and the variables each tool measures.

**Table 1: Study measurement tools, including method and time frame for their use and what variables they measure.**

| Tool | Method | Time Frame | Variables Measured |
|---|---|---|---|
| Core Tool (modified)<br><br>(appendix 4) | Core tool completed by level 1 trained lymphoedema physiotherapist at initial appointment.<br><br>The core tool can be completed using information from the patient and/or their family or from the patient's medical record. | The core tool will be completed at the initial assessment | Patient demographics:<br>- Age<br>- Gender<br>- *Level of obesity*<br>- Relevant comorbidities<br>- *History of fungal infections/tinea between toes*<br>- Duration of oedema<br>- *Use of prophylactic antibiotics*<br>- *History of cellulitis*<br>- Referral source |
| Cellulitis Tool<br><br>(appendix 4) | Self-report of if cellulitis has recurred post intervention, the date it recurred and if hospitalisation resulted will be gained at regular scheduled appointments by a therapist or via phone interview. Date of recurrence of cellulitis may be checked or confirmed through each patient's general practitioner or medical record (from Calvary Public Hospital Bruce and Canberra Hospital and Health Services). | The cellulitis tool will be completed every 6 months post initial intervention until the study finishes. | - Date of initial intervention<br>-Date last contact was made with patient<br>**- Time to first episode of recurrent cellulitis post intervention (days)**<br>**- If participant was hospitalised for episode of cellulitis (+ date of hospitalisation)**<br>- Adverse events |

| | | | |
|---|---|---|---|
| | | | -*Occurrence of tinea/fungal infections*<br>-*Occurrence of a wound*<br>-Garment compliance |
| LYMQOL (appendix 4) | LYMQOL questionnaire will be completed by the patient where possible. | LYMQOL will be completed at initial assessment and then 6, 12, 24 and 36 months post intervention. | **- QOL** |
| Perometer (appendix 4) | A qualified level one lymphoedema therapist will conduct perometry measures as per standard measurement protocol. | Perometry will be conducted prior to intervention, immediately post intervention and then 6,12, 18, 24, 30 and 36 months post intervention.<br>(perometry measures will be recorded on the cellulitis tool) | **- Absolute and percent change in volume of leg oedema (ml)** |

Main outcome factors have been bolded. Identified potential confounding factors have been italicised.

**Data Analysis**

For the main outcome measure of 'time to first episode of recurrent cellulitis', survival analysis will be undertaken. Kaplan-Meier plots will be used to compare the time to first recurrence between the groups, with a log rank test being used to determine if there is a statistically significant difference between the groups. Cox proportional hazards regression may also be used to adjust for important risk factors. Right censoring will be used for patients who are lost to follow up.

For the secondary outcomes of 'percent change in limb volume' and 'QOL', measures will be at multiple time points. Therefore groups may be compared using a linear mixed model or using a repeated measures analysis. Intention to treat analysis will be used.

Data will be entered into excel spreadsheets in a form appropriate for statistical analysis. Analysis of data will be performed using STATA software. Guidance regarding statistical analysis will be gained through Dr Terry Neeman and Jiali Wang from the statistical consulting unit at the Australia National University.

**Ethical Concerns**

This research will be conducted in accordance with the National Statement on Ethical Conduct in Human Research (National Health and Medical Research Council [NHMRC], 2007). Application for ethical approval will be submitted to the *CPHB* and ACT HEALTH Human Research Ethics Committees (HREC). Patient confidentiality and secure storage of data will be maintained as per CPHB regulations.

Each participant will be fully informed of the nature and scope of their involvement in the study and will be provided with an opportunity to ask questions prior to providing consent. Participants will be made aware that they can freely decline or withdraw participation in the study at any point and this will have no impact to their ongoing access to health services. Patients will be informed that information regarding cellulitis recurrence may be gained from their medical (from Calvary Public Hospital Bruce and Canberra Hospital and Health Services) or GP to verify self-reported incidence or gain data if they drop out or withdraw from the study, unless requested otherwise.

There are no risks to patients participating in the study that are additional to the standard risks that occur with compression therapy. Standard risks of compression therapy include development of pressure/tissue injury, reaction to compression material, arterial compromise and it's sequela, discomfort, financial harm (if purchased compression is not beneficial), psychological distress and increased risk of falling whilst wearing compression bandages. These risks are low and will be

minimised through patient education and thorough assessment and appropriate treatment prescription from trained lymphoedema therapists. Knowledge gained through this research may contribute to improved care of this patient population in the future. Both the intervention and control groups will receive and potentially benefit from free compression. Patients will be informed of the identified potential risks and benefits of participating in the study. In-line with the National Statement of Ethical Conduct in Human Research, risks and unanticipated or adverse outcomes will be monitored and if we find the research may be compromising patient welfare, the HREC will be contacted to investigate if ethical approval should be withdrawn (NHMRC, 2007).

The absence of high quality evidence regarding the impact of compression therapy on recurrence of cellulitis means there is uncertainly in which study group will fare better, justifying use of an RCT to conduct further research. Whilst there is no high quality evidence to support use of the compression therapy in this patient population, it is our standard clinical practice within our institution (this is not so in other settings). Therefore we have designed the RCT with cross-over of the control group, so that patients in this group do not continue to experience episodes of recurrent cellulitis without receiving the intervention we believe is effective.

**Proposed Research Timeline**

| | **2016** | | | **2017** | | | **2018** | **2019** | **2020** | **2021** | **2022** |
|---|---|---|---|---|---|---|---|---|---|---|---|
| | O | N | D | J | F | Mar to Dec | | | | | |
| Formalise funding agreement for supply of compression garments | ■ | | | | | | | | | | |
| Finalise proposal | ■ | ■ | | | | | | | | | |
| Submit NEAF to HRECs (with relevant consent and patient information forms) | | ■ | | | | | | | | | |
| Ethics approval from both hospitals involved | | | ■ | ■ | | | | | | | |
| GPs and hospital staff educated about study and referral processes (ongoing) | | | | | ■ | ■ | ■ | | | | |
| Referral forms disseminated | | | | | ■ | ■ | ■ | | | | |
| Staff trained in use of outcome measures | | | | ■ | | | | | | | |
| Refresher staff training regarding outcome measures | | | | | | | ■ | ■ | ■ | ■ | |
| Patient recruitment | | | | | ■ | ■ | ■ | | | | |
| Data Collection | | | | | ■ | ■ | ■ | ■ | ■ | ■ | ■ |

| | | | | | | | | | | | |
|---|---|---|---|---|---|---|---|---|---|---|---|
| Data Analysis | | | | | | | | | | | |
| Complete report and prepare for publication | | | | | | | | | | | |
| Disseminate and discuss results with stakeholders | | | | | | | | | | | |

**Stakeholder involvement**

For this study to be successful, support of key stakeholders to refer appropriate patients is integral. Staff managing hospital wards known to see a high proportion of cellulitis cases have been contacted and involved in planning of appropriate referral pathways and tools. Clinicians who have primary involvement in management of patients with acute cellulitis and therefore the ability to refer them on, are major stakeholders. Therefore extensive education in written (newsletters, handouts and posters) and verbal (education sessions, GP information evenings) form will directed to clinicians at involved hospitals, and within GP practices. Throughout the study, regular contact will be made with clinicians on target wards to encourage referrals and provide feedback regarding study progress.

**Dissemination strategy**

The study findings will be summarised in an article that will be submitted to a relevant peer-reviewed journal. We plan to disseminate our findings to key stakeholders, including primary contact clinicians for patients experiencing cellulitis (doctors and health professionals in acute and community settings), clinicians who manage chronic oedema and professionals who may be involved in developing relevant policy and practice. At a local level, results may be presented at grand rounds and GP education evenings and provided to study participants and local interest groups (e.g. ACT Lymphoedema support group) in lay form. We plan to present findings at relevant conferences and webinars (e.g. International Lymphoedema Framework conference) and on websites of leading body associations (e.g. Australasian Lymphology Association).

**Importance of this research:**

In major Australian public hospitals from 2011-2012 there were 23,432 admissions for cellulitis without complications, with the average admission lasting 3.8 days and costing $3,800 (National Health Performance Authority [NHPA], 2015). A high proportion of individuals who develop cellulitis will go on to experience recurrence; recurrence has been reported to vary between 29-47% within a three-year time frame in international populations (Cox, 2006; Jorup-Ronstrom & Britton, 1987). Therefore it can be assumed that a significant number of reported annual admissions for cellulitis are for recurrent episodes.

Knowledge that chronic oedema increases risk of cellulitis is now particularly pertinent, as changes in the Australian population demographic suggest that chronic oedema is likely becoming increasingly common. Chronic oedema is associated with obesity, cancer survivorship, old age and likely poor mobility (Moffatt et al., 2004; Boza et al., 2011; Graf et al., 2013; Solaro et al., 2006), all of which are known to be increasingly common within Australia (Phillips & Currow, 2010; Cameron et al., 2003; Australian Institute of Health and Welfare [AIHW], 2014). Increasing chronic oedema prevalence will result in a larger population having increased risk of cellulitis. As cellulitis negatively impacts QOL of sufferers and is a cause of financial burden for individuals and the healthcare system system (Al-Niaimi & Cox, 2013; Carter, Kilburn, & Featherson, 2007), the increase in chronic oedema represents a growing public health issue that needs to be addressed.

The time intensive nature of compression management and the fact the measurable outcomes require lengthy assessment periods, contribute to the lack of research in this field. Currently two studies only have been conducted on the impact of compression management on cellulitis recurrence (Arsenault et al., 2011; Ko et al., 1998), with no research on this area being performed within Australia. As both studies use pre-post intervention methods and have either a small sample size or only assess cellulitis as a secondary outcome, generating only low-level evidence (Arsenault et al., 2011: Ko et al., 1998), further research in this area is warranted. The lack of evidence regarding the impact of compression to manage chronic oedema on cellulitis makes it challenging to justify clinical services, determine treatment priorities and realise potential financial benefits. If this research shows that compression increases time to cellulitis recurrence, it may be used to justify greater investment in provision of compression therapy as this may decrease the overall cost to the healthcare system and reduce financial and personal burden of sufferers through decreased number of cellulitis episodes and hospital admissions.

**Reference list:**

Al-Niaimi, F., Cox, N. (2009). Cellulitis and lymphoedema: a vicious cycle. *Journal of Lymphoedema, 4*(2), 38-42.

Arsenault, K., Rielly, L., Wise, H. (2011). Effects of Complete Decongestive Therapy on the Incidence Rate of Hospitalization for the Management of Recurrent Cellulitis in Adults with Lymphedema. *Rehabilitation Oncology, 29*(3), 14-20. Retrieved from

http://search.proquest.com.wwwproxy0.library.unsw.edu.au/docview/912510464?accountid=12763&rfr_id=info%3Axri%2Fsid%3Aprimo.

Australian Institute of Health and Welfare (AIHW). (2014). Healthy life expectancy in Australia: patterns and trends 1998 to 2012. Canberra, Australia: Australian Institute of Health and Welfare. Retrieved from: http://www.aihw.gov.au/publication-detail/?id=60129549634.

Boza, J. C., Trindade, E. N., Peruzzo, J., Sachet, L., Rech, L., Cestari, T. F. (2011). Skin manifestations of obesity: a comparative study. Journal of the European Academy of Dematology and Venereology. *Journal of the European Academy of Dermatology and Venereology*, *26*(10), 1220-23.

Bulley, C., Coutts, F., Tan, C. W. (2013). Perometry limb volume measurement: protocol development and reliability. *European Journal of Physiotherapy, 15*(4), 193-200. doi: 10.3109/21679169.2013.831482.

Cameron, A. J., Zimmet, P. Z., Dunstan, D. W., Dalton, M., Shaw, J. E., Welborn, T., . . . Jolley, D. (2003). Overweight and obesity in Australia: the 1999–2000 Australian Diabetes, Obesity and Lifestyle Study (AusDiab). *The Medical Journal of Australia, 178*(9), 427-432.

Carter, K., Kilburn, S., Featherston, P. (2007). Cellulitis and treatment: a qualitative study of experiences. *British Journal of Nursing, 16*(6), 22-4, 26-8. doi:10.12968/bjon.2007.16.Sup1.27089

Chlebicki, M. P., & Oh, C. C. (2014). Recurrent Cellulitis: Risk Factors, Etiology, Pathogenesis and Treatment. *Current Infectious Disease Report, 16*(9), 422. doi: 10.1007/s11908-014-0422.

Cox, N. H. (2006). Oedema as a risk factor for multiple episodes of cellulitis/erysipelas of the lower leg: a series with community follow-up. *The British Journal of Dermatology, 155*(5), 947-50. doi: 10.1111/j.1365-2133.2006.07419.x.

Cox, N. H., Colver, G. B., Paterson, W. D. (1998); Management and morbidity of cellulitis of the leg. *Journal of the Royal Society of Medicine, 91*(12), 634-7. Retrieved from http://www.ncbi.nlm.nih.gov/pmc/articles/PMC1296982/pdf/ jrsocmed00018-0028.pdf.

Dupuy, A., Benchikhi, H., Roujeau, J. C., Bernard, P., Vaillant, L., Chosidow, O…. Bastuji-Garin, S. (1999). Risk factors for erysipelas of the leg (cellulitis): case control study. *British Medical Journal, 318*(7198), 1591-4. Retrieved from

http://search.proquest.com.wwwproxy0.library.unsw.edu.au/docview/1777603258?OpenUrlRefId=info:xri/sid:primo&accountid=12763.

Graf, N., Rufibach, K., Schmidt, A. M., Fehr, M., Fink, M., Baege, A. C. (2013). Frequency and risk factors of lower limb lymphoedema following lymphadenectomy in patients with gynaecological malignancies. *European Journal of Gynecological Oncology*, *34*(1), 23-7.

Haesler, E. (2015). Evidence Summary Lymphoedema: Objective assessment using perometry. *Wound Practice and Research*, *23*(2), 94-95.

Inghammar, M., Rasmussen, M., Linder, A. (2014) Recurrent Erysipelas- risk factors and clinical presentation. *BMC Infectious Diseases*,*14*, 270. Retrieved from: http://www.biomedcentral.com/1471-2334/14/270.

Jorup-Ronstrom, C., & Britton, S. (1987). Recurrent erysipelas: predisposing factors and costs of prophylaxis. *Infection*, *15*(2),105–6. doi:10.1007/BF0165020.

Keeley, V., Crooks, S., Locke, J., Veigas, D., Riches, K., Hilliam, R. (2010). A quality of life measure for limb lymphoedema (LYMQOL*). Journal of Lymphoedema*, *5*(1), 26–37. Available from http://oro.open.ac.uk/32567/4/JoL_Quality_of_Life_Measures.pdf.

Ko, D. S., Lerner, R., Klose, G., Cosimi, A. B. (1998). Effective treatment of lymphedema of the extremities. *Archives of Surgery*, *133*(4), 452-8.

Moffatt, C. J., Franks, P. J., Doherty, D. C., Williams, A. F., Badger, C., Jeffs, E, . . . Mortimer, P. S. (2003). Lymphoedema: an underestimated health problem. *Monthly journal of the association of physicians*, *96*, 731-38.

Mortimer, P. S. (2000). ABC of arterial and venous disease: Swollen lower limb – 2: Lymphoedema. *British Medical Journal*, *320*(3), 1527-1529.

Morton, N., & Swartz, M. N. (2004). Cellulitis. *New England Journal of Medicine*, *350*, 904-12. doi: 10.1056/NEJMcp031807.

National Health and Medical Research Council (NHMRC). (2007). National Statement on Ethical Conduct in Human Research 2007 (Updated May 2015). Canberra, Australia: National Health and Medical Research Council. Available from: www.nhmrc.gov.au/guidelines/publications/e72

National Health Performance Authority. (2015). Hospital Performance: Costs of acute admitted patients in public hospitals in 2011–12. Sydney, Australia: National Health Performance Authority. Retrieved from: https://www.myhospitals.gov.au/docs/default-source/our-report-pdfs/hp_costsofacuteadmittedpatients_2011_12_rpt.pdf?sfvrsn=18

Perdomo, M., Davies, C., Levenhagen, K., Ryans, K. (2014). Assessment Measures of Secondary Lymphedema in Breast Cancer Survivors. *Rehabilitation Oncology, 32*(1), 22-35.

Phillips, J. L., Currow, D. C. (2010). Cancer as a chronic disease. *Collegian, 17*(2), 47-50. doi: http://dx.doi.org/10.1016/j.colegn.2010.04.007.

Solaro, C., Uccelli, M. M., Brichetto, G., Augello, G., Taddei, G., Boccardo, F., . . . Mancardi, G. L. (2006). Prevalence of oedema of the lower limb in multiple sclerosis patients: a vascular and lymphoscintigraphic study. *Multiple Sclerosis, 2*, 659-61.

## Appendixes

### Appendix 1: Definition of key terms

Cellulitis: Cellulitis is a common acute bacterial infection of the subcutaneous tissue and skin (Morton & Swartz, 2004). Erysipelas is an infection similar to cellulitis, which typically affects more superficial tissues. For the purpose of our study, we will consider erysipelas and cellulitis as one entity as the terms are often used interchangeably and most clinical studies and data do not differentiate between them (Chlebicki & Oh, 2014).

Chronic oedema: Chronic oedema is persistent swelling that has been present for a minimum of three months and does not resolve with elevation (Moffatt et al., 2004). The umbrella term of chronic oedema is now often used instead of lymphoedema, as it is considered to include all people with oedema present for three or more months, regardless of the cause (Mortimer, 2000).

Compression therapy**:** Compression therapy will consist of prescription of appropriate compression garments and where indicated provision of a period of compression bandaging to minimise swelling

prior to measure for and fit of compression garments. Compression garments with or without bandaging will be renewed regularly (every 6-12 months).

**Appendix 2: Eligibility criteria for participants**

Table 2: Eligibility criteria for participants

| Inclusion Criteria: | Exclusion Criteria |
|---|---|
| • The patients is over 18 years of age<br>• The patient is identified as having chronic oedema (oedema persisting more than 3 months) in one or both legs<br>• The patient has a history of 2 or more episodes of cellulitis in the 2years prior to trial referral<br>• The patient is able to understand their involvement in the study, as per the information sheet<br>• The patient is able to give informed consent<br>• The patient has a valid Medicare card<br>• The patient is able to attend regular scheduled appointments | • The patient is currently receiving chronic oedema management that involves compression<br>• The patient declines to participate or is unable to participate for whatever reason<br>• The patient is receiving end of life care<br>• The patient is medically unstable<br>• Chronic wound/ulcer, or a wound/ulcer requiring specialist treatment or treatment that prevents the use of compression garments.<br>• The patient is unable to wear compression (unable to don/doff garments or has a medical condition that contraindicates use of compression) |

**Appendix 3: Participant randomisation process.**

Figure 1: Participant randomisation process

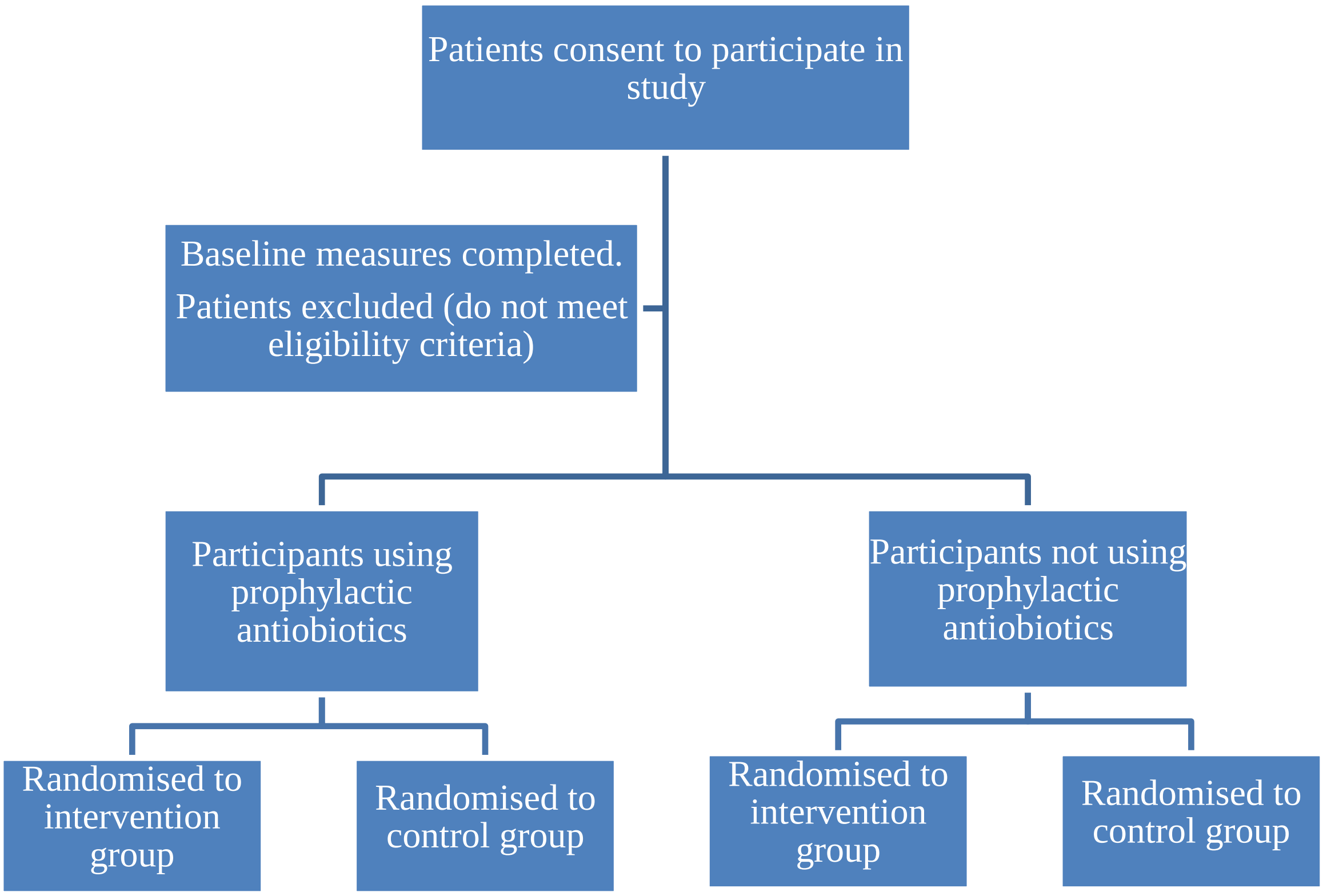


**Appendix 4: Measurement Tools**

<u>LYMQOL:</u> LYMQOL is a validated quality of life tool for people with lower limb lymphoedema (Keeley et al., 2010).

<u>Perometer:</u> A perometer is an optoelectronic imaging device that can measure limb volume (Bulley, Coutts, & Tan, 2013). The perometer has been found to be a reliable (intra-rater and inter-rater) measurement device that is sensitive to change in limb volume (Bulley et al., 2013; Haesler, 2015; Perdomo, Davies, Levenhagen, & Ryans, 2014).

BMJ Open

# Impact of Compression Therapy on Cellulitis (ICTOC) in adults with chronic oedema: a randomised controlled trial protocol

Elizabeth Webb,[1,2] Teresa Neeman,[3] Jamie Gaida,[4] Francis J Bowden,[5,6] Virginia Mumford,[7] Bernie Bissett[2]

**To cite:** Webb E, Neeman T, Gaida J, *et al*. Impact of Compression Therapy on Cellulitis (ICTOC) in adults with chronic oedema: a randomised controlled trial protocol. *BMJ Open* 2019;**9**:e029225. doi:10.1136/bmjopen-2019-029225

► Prepublication history for this paper is available online. To view these files, please visit the journal online (http://dx.doi.org/10.1136/bmjopen-2019-029225).

Received 17 January 2019
Revised 30 June 2019
Accepted 3 July 2019

Check for updates

© Author(s) (or their employer(s)) 2019. Re-use permitted under CC BY-NC. No commercial re-use. See rights and permissions. Published by BMJ.

For numbered affiliations see end of article.

**Correspondence to**
Elizabeth Webb;
elizabeth.webb@calvary-act.com.au

## ABSTRACT

**Introduction** Cellulitis represents a significant burden to patients' quality of life (QOL) and cost to the healthcare system, especially due to its recurrent nature. Chronic oedema is a strong risk factor for both an initial episode of cellulitis and cellulitis recurrence. Expert consensus advises compression therapy to prevent cellulitis recurrence in individuals with chronic oedema, however, there is little supporting evidence. This research aims to determine if the management of chronic oedema using compression therapy effectively delays the recurrence of lower limb cellulitis.

**Methods and analysis** A randomised controlled trial with cross-over will be used to assess the impact of compression therapy on clinical outcomes (time to next episode of cellulitis, rate of cellulitis-related hospital presentations, QOL and leg volume). Using concealed allocation, 162 participants will be randomised into the intervention (compression) or control (no compression) group. Randomisation will be stratified by prophylactic antibiotic use. Participants will be followed up at 6 monthly intervals for up to 3 years or until 45 episodes of cellulitis occur across the cohort. Following an episode of recurrent cellulitis, control group participants will cross-over to the intervention group. Survival analysis will be undertaken to assess the primary outcome measure of time to cellulitis recurrence. The hypotheses are that compression therapy to control lower limb chronic oedema will delay recurrent lower limb cellulitis, reduce the rate of associated hospitalisations, minimise affected limb volume and improve the QOL of this population.

**Ethics and dissemination** Ethics approval has been obtained from the ethics committees of all relevant institutions. Results will be disseminated through relevant peer-reviewed journal articles and conference presentations.

**Trial registration number** ACTRN12617000412336; Pre-results. The ICTOC trial is currently in progress. Participant recruitment started in May 2017 and is expected to continue until December 2019.

## Strengths and limitations of this study

- Randomisation of participants will be stratified by prophylactic antibiotic use to ensure antibiotic use does not confound treatment outcome.
- Due to the nature of the intervention, blinding is not feasible for participants or assessors.
- Assessment tools and methods (perometer, diagnosis of cellulitis by medical practitioners external to the trial, verification of data using the medical record or general practitioner) have been selected to minimise potential measurement bias.
- The use of broad inclusion criteria will allow for trial results to be generalised to adults across a range of settings nationally and internationally.

## BACKGROUND AND RATIONALE

Cellulitis is a common acute bacterial infection of the skin and subcutaneous tissue.[1] The majority of cellulitis episodes (69%–81%) occur in the lower limbs.[2–4] In Australia, lower limb cellulitis is associated with significant health costs due to frequent hospital admissions and high levels of morbidity. In 2014–2015, there were 59 466 hospitalisations for cellulitis,[5] with the average admission lasting 4.3 days.[6] In 2013–2014, cellulitis was the third leading cause of potentially preventable hospital admissions, with over half of all admissions for cellulitis being considered potentially preventable.[6 7] Erysipelas is an infection similar to cellulitis, which typically affects more superficial tissues. As the terms erysipelas and cellulitis are often used interchangeably and most clinical studies do not differentiate between them, this paper will consider them as one entity.

Recurrence of cellulitis is common and represents a significant proportion of the disease burden. In a 3-year time frame cellulitis has been reported to recur in 29%–47% of patients,[8 9] with a case series in Sweden finding that 13% of patients admitted for cellulitis developed two or more recurrences within 3 years.[9] In light of the significant recurrence rates, effective interventions

BMJ Open: first published as 10.1136/bmjopen-2019-029225 on 15 August 2019. Downloaded from http://bmjopen.bmj.com/ on August 22, 2019 by guest. Protected by copyright.

which reduce recurrence could limit the disease burden and improve patient outcomes.

Oedema occurs when capillary filtration overwhelms the available lymphatic drainage.[10] Lymphoedema specifically refers to persistent oedema resulting from lymphatic drainage failure.[11] Chronic oedema is an umbrella term that refers to oedema resulting from insufficient lymphatic drainage, where the principal cause of oedema may be increased capillary filtration and/or lymphatic drainage failure.[11] As such, the term chronic oedema encompasses oedema of various aetiologies, including lymphoedema. For the purpose of this trial, we will use the term chronic oedema.

Lymphoedema and chronic oedema are potent risk factors for developing lower limb cellulitis and for its recurrence.[4 8 12 13] It is broadly accepted that the relationship between cellulitis and chronic oedema is a vicious cycle.[8 14] Chronic oedema predisposes individuals to cellulitis and with each episode of cellulitis, the lymphatic system is further impaired, increasing residual oedema and heightening risk of future cellulitis infections.[14] Thus, chronic oedema is not only a result of cellulitis but also increases the risk of recurrence.[14]

The standard treatment for chronic oedema includes compression therapy and skin care.[15] Compression bandaging can be used to reduce oedema in a limb, and daily wear of compression garments is used to control oedema. There is general consensus that in addition to antibiotic prescription, compression to manage oedema should be an adjuvant treatment for patients with chronic oedema who are experiencing cellulitis recurrence.[1 8 14 16] Despite this common recommendation and the strong evidence supporting the relationship between oedema and cellulitis, there is a paucity of evidence to support the use of compression to manage chronic oedema to prevent cellulitis recurrence.

The time-intensive nature of compression therapy and the fact that measuring meaningful outcomes requires lengthy assessment periods probably contribute to the lack of research in this field. Only one study has been conducted on the impact of oedema management on cellulitis recurrence,[17] with a second study incidentally observing a reduction in 'infection' among patients receiving oedema management, although this was not a research objective.[18] While both studies support the hypothesis that oedema management decreases cellulitis recurrence, their conclusions are hampered by methodological limitations, including pre-post intervention methods, small sample sizes and change in infection rate not being specified a research objective.[17 18] While research regarding compression therapy to prevent cellulitis recurrence is scarce, there is high-quality evidence to support the use of prophylactic antibiotics. A multicentre, double-blind, randomised controlled trial (RCT) found that the use of prophylactic antibiotics in patients experiencing recurrent cellulitis is effective in preventing subsequent attacks, although the effect diminishes following prophylaxis cessation.[19] A 2017 Cochrane systematic review of interventions to prevent cellulitis identified six studies investigating prophylactic antibiotics, but no other randomised trials investigating other prophylactic measures such as oedema management or skin care.[20] Thus further research into the efficacy of prophylactic measures other than antibiotic is warranted.[20]

The following protocol describes a RCT with cross-over to determine if the use of compression therapy for adults experiencing lower limb recurrent cellulitis and chronic oedema will delay cellulitis recurrence.

## RESEARCH HYPOTHESES

The hypotheses are that compression therapy to control lower limb chronic oedema will delay recurrent lower limb cellulitis, reduce the rate of associated hospitalisations, minimise affected limb volume and improve the quality of life (QOL) of this population.

## RESEARCH OBJECTIVES

### Primary objective

The primary objective was to determine if compression therapy delays the recurrence of lower limb cellulitis in adults with lower limb chronic oedema and recurrent cellulitis.

### Secondary objectives

The secondary objectives were to determine if, in adults with lower limb chronic oedema and recurrent cellulitis, compression therapy: (1) reduces the rate of cellulitis-related hospital presentations; (2) reduces affected leg volume; and (3) improves QOL.

## TRIAL DESIGN

A RCT with cross-over will be used to assess the impact of compression therapy on clinical outcomes (time to next episode of cellulitis, rate of cellulitis-related hospital presentations, QOL and leg volume). Participants will be randomised to the intervention or control group by block randomisation using sealed opaque envelopes. As prophylactic antibiotics have been shown to influence cellulitis recurrence,[19–21] randomisation of participants will be stratified by prophylactic antibiotic use. Following an episode of cellulitis, participants in the control group will cross-over into the intervention group, whereas intervention group participants will remain in their original group and continue to receive compression therapy. Figure 1 shows the proposed participant allocation process.

The absence of high-quality evidence regarding the impact of compression therapy on recurrence of cellulitis means there is uncertainty as to whether it is an effective intervention, justifying the use of an RCT. Although there is no high-quality evidence to support the use of the compression therapy to prevent cellulitis in this patient population, it reflects the accepted expert opinion and the standard clinical practice of the institution conducting the trial.

BMJ Open: first published as 10.1136/bmjopen-2019-029225 on 15 August 2019. Downloaded from http://bmjopen.bmj.com/ on August 22, 2019 by guest. Protected by copyright.

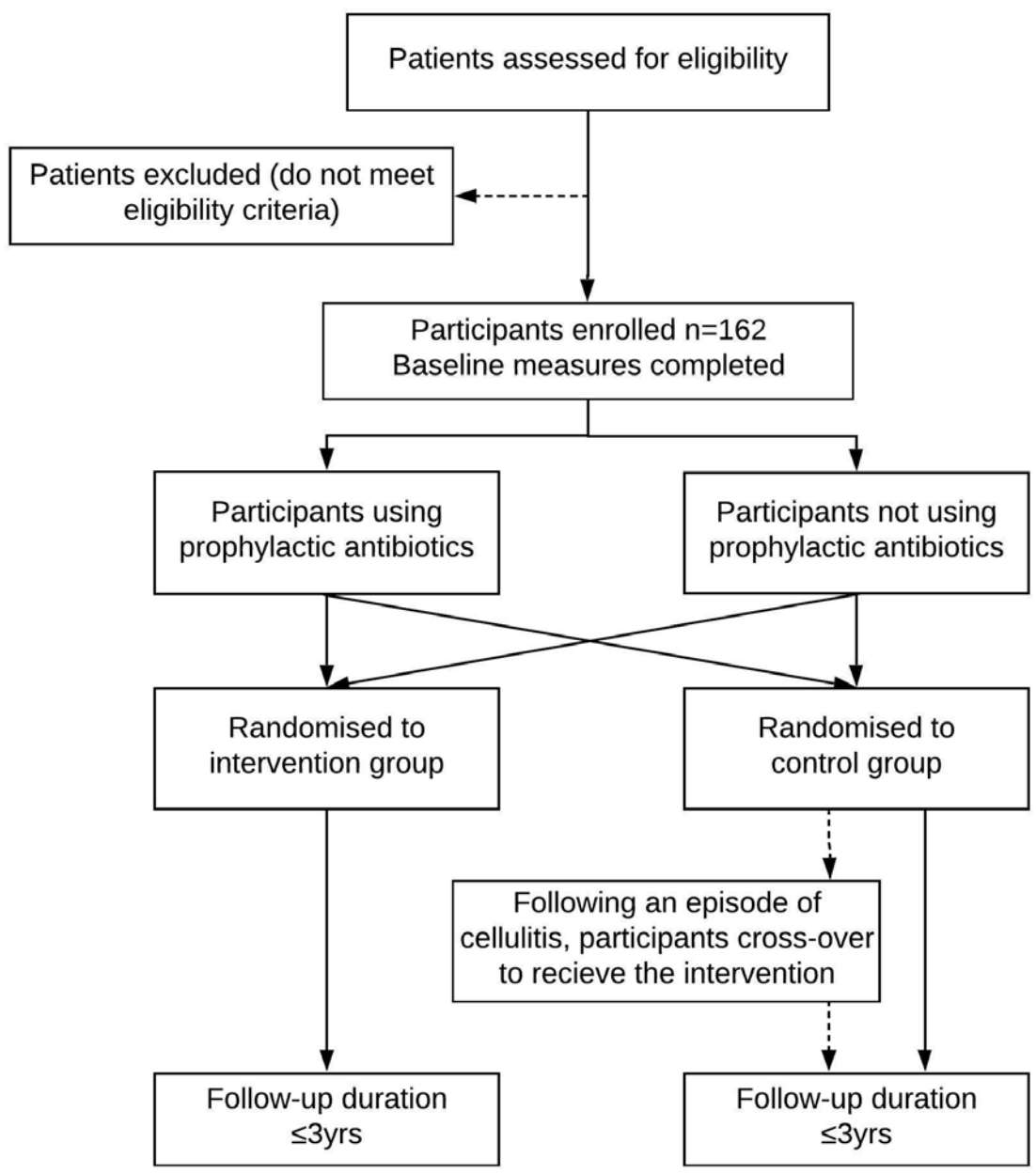


**Figure 1** Anticipated participant flow through trial.

Therefore, the trial design crosses the control group participants over into the intervention group following the first episode of cellulitis to ensure no participant continues to experience recurrent cellulitis episodes without receiving the institution's standard intervention.

## METHODS

### Study setting and population

The trial will be conducted at the Calvary Public Hospital Bruce (CPHB) outpatient lymphoedema clinic. Adults with lower limb chronic oedema and a history of recurrent cellulitis who meet the eligibility criteria will be recruited from the two major ACT public hospitals (CPHB and Canberra Hospital) and general practitioners servicing the ACT and nearby NSW residents.

### Eligibility criteria

#### Inclusion criteria

- ≥ 18 years of age.
- Two or more episodes of cellulitis diagnosed in the same leg in the past 2 years (at the time of referral). Clinical diagnosis of cellulitis ideally will have been based on the presence of acute erythema, oedema, warmth and pain, with spreading involvement of the skin and subcutaneous tissues, malaise and possibly fever.[1 22 23]
- Chronic oedema (oedema persisting ≥3 months) in the leg(s) that have had recurrent cellulitis diagnosed (presence of oedema confirmed by an accredited lymphoedema therapist through interview and physical examination, including a thorough medical history combined with limb palpation and visual assessment).
- Understanding of involvement in the study as per the participant information sheet.
- Provision of informed consent.
- Able to attend regularly scheduled appointments for the duration of the study.
- Has a valid Medicare number.

#### Exclusion criteria

- Currently wearing effective compression garments (≥compression class 2, or compression class 1 if considered effective by a lymphoedema therapist) regularly (≥5 days per week).
- Declines to participate or is unable to participate for whatever reason.
- Receiving end of life care.
- Medically unstable.
- Chronic wound/ulcer, or a wound/ulcer requiring specialist treatment or treatment that prevents the use of compression garments.
- Unable to wear compression (unable to don/doff garments or has a medical condition that contraindicates the use of compression).

### Interventions

All assessments, interventions and outcome measures will be conducted by a physiotherapist or occupational therapist who meets the registration requirements for category one of the Australian National Lymphoedema Practitioners Register.[24]

At the initial appointment, eligibility will be formally checked, and those who consent to participate will undergo stratified randomisation using sealed, opaque and identical envelopes that are sequentially numbered. Prior to randomisation, baseline measures including number of episodes of cellulitis in the 2 years prior to referral, duration of chronic oedema, referral source and demographics will be captured. The presence of identified potential risk factors for cellulitis will also be recorded, including history of tinea or other fungal infections between toes, diabetes mellitus, obesity and chronic venous insufficiency.[3 4 12 25 26]

At the initial appointment, participants in both the control and intervention groups will receive education (verbal and written) regarding cellulitis and how to decrease the risk of recurrence. Education will include the benefits of skin care, prevention of tinea or other fungal infections between toes, maintaining healthy body weight and regular exercise.

For the intervention group, the initial appointment will also be used to plan appropriate compression therapy which will be provided at subsequent appointments. Compression therapy will involve the application of compression garments (compression stockings or wraps) and may or may not involve compression bandaging to minimise oedema prior to the fitting of compression garments. The number of appointments necessary for the provision of compression therapy will be individualised to meet participant requirements.

BMJ Open: first published as 10.1136/bmjopen-2019-029225 on 15 August 2019. Downloaded from http://bmjopen.bmj.com/ on August 22, 2019 by guest. Protected by copyright.

Participants in both groups will be followed for up to 3 years at 6 monthly intervals (table 1) to complete outcome measures and to continue to receive the allocated treatment (education with or without compression therapy). At each appointment, the therapist will inform each participant of changes in their limb volume, providing tangible feedback to support ongoing participant attendance. Throughout the trial, participants in the intervention group may require additional appointments for compression therapy (compression bandaging, and measure for and provision of compression garments). Intervention compliance (number of days per week garments are worn) and adverse effects will be captured by self-report.

Cross-over of control group participants will be triggered on clinician identification of cellulitis. The recurrence of cellulitis will be checked at scheduled appointments, however, if a participant reports a recurrence between scheduled assessments, they will be reviewed at an additional appointment to record outcome measures (table 1), and to commence cross-over for control group participants. Date of cross-over will be defined as the day compression garments are initially fitted.

### Outcome measures

Table 1 shows the timeline for completion of trial activities and outcome measures.

The primary outcome is 'observed time to the first episode of cellulitis recurrence'. Cellulitis recurrence will only be assessed in a leg that has been assessed as having chronic oedema, thus if cellulitis occurs in a leg that was not previously identified as having chronic oedema, the infection will not be considered a recurrence. Cellulitis will be diagnosed by medical practitioners external to the study. Date of cellulitis recurrence (and associated hospitalisation) will be gained by participant self-report and may be verified using medical records from the hospitals and/or general practitioners.

Secondary outcomes include: (1) rate of cellulitis-related hospital admissions; (2) percent change in leg volume from baseline, measured using the perometer; (3) QOL, assessed using the LYMQOL and EuroQol Five Dimension Scale (ED-5D-3L). The occurrence of cellulitis-related hospital admissions will be measured in the same manner as cellulitis recurrence.

Percent change in leg volume will be measured using a perometer, an optoelectronic imaging device designed to measure limb volume.[27] The perometer has excellent intra-rater reliability (ICC=1.0; 95% CI: 0.99 to 1.00) and inter-rater reliability (ICC=1.0; 95% CI: 0.97 to 1.00), is sensitive to changes in limb volume[28–30] and is a valid measure of knee volume.[27] Leg volume will be measured between 53 and 400 mm height from the ground using the perometer. Monthly calibration of the perometer will be conducted using a standardised object of known volume (875 mL) to minimise instrument error, ensuring consistency of this measurement device across the duration of the trial. Use of this device will also prevent potential differential measurement bias arising from lack of therapist blinding.

Where limb volume cannot be measured using the perometer, due to impaired mobility of a participant or equipment failure, summated circumferential leg measurements will be used following expert clinical guidelines. Circumferential leg measures will be taken at the mid foot, oblique ankle and at 10, 20, 30 and 40 cm intervals up the leg using a measurement board. Circumferential limb measurement also has excellent intra-rater reliability (ICC=0.977–0.996; 95% CI: 0.960 to 0.998) and inter-rater reliability (ICC=0.942–0.994; 95% CI: 0.936 to 0.997).[31]

QOL will be measured using LYMQOL, a validated, condition-specific QOL tool for people with lower limb lymphoedema,[32] and the EQ-5D-3L, a generic preference-based measure of health-related QOL that comprises five dimensions of health.[33] The EQ-5D can be used to calculate quality-adjusted life years for the purpose of economic evaluation.[33] A systematic review has found the EQ-5D has good validity and responsiveness for people with skin diseases, although the tool has not been specifically validated within a population suffering cellulitis.[33]

Exploratory analysis will be conducted to test the robustness of the trial hypotheses and may include assessment of cellulitis recurrence post cross-over, intervention compliance, participant demographics, risk factors and per protocol analysis.

### Sample size and duration of follow-up

The sample size has been calculated for the primary objective of detecting a difference in time to cellulitis recurrence between the control and intervention groups. The sample size estimation is based on the assumptions that the 3-year cellulitis recurrence rate in control participants is approximately 47%[8] and compression therapy will reduce the 3-year incidence of recurrent cellulitis by 50%.[17 18] Assuming that events occur at a constant rate, these assumptions correspond to a hazard ratio (HR) of 0.42. The eligibility criteria of two or more episodes of cellulitis in the same leg in the past 2 years has been used so that the trial cohort have an increased likelihood of cellulitis reoccurring during the follow-up period.

It is assumed that patients will be recruited over a 2.5 year period, and the total study duration will be 3.5 years. Length of participant follow-up will vary based on time of enrolment. Using a sequential design software package gsDesign in R,[34] in order to detect a HR of 0.42 with 80% power and 2.5% (one-sided) type 1 error, a total of 45 cellulitis recurrences are needed. Under the present recruitment and recurrence assumptions, we plan to recruit 162 participants (81 per arm).

An interim analysis will be performed by a Data Monitoring Committee after 23 episodes of cellulitis. A log-rank test will be used to assess group differences. If a nominal (one sided) significance level of p=0.003 is detected, indicating a strong clinical effect, the study will be ceased. If the Data Monitoring Committee recommends that the study continue to 45 episodes of cellulitis,

BMJ Open: first published as 10.1136/bmjopen-2019-029225 on 15 August 2019. Downloaded from http://bmjopen.bmj.com/ on August 22, 2019 by guest. Protected by copyright.

**Table 1** Timeline per patient for RCT outcome measures

| Time point | Enrolment | Assessment post initial intervention | Assessment post cellulitis recurrence | Cross -over | 6 | 12 | 18 | 24 | 30 | 36 |
|---|---|---|---|---|---|---|---|---|---|---|
| *Body mass index* | X | | X | X | X | X | X | X | X | X |
| Perometer (limb volume) | X | X | X | X | X | X | X | X | X | X |
| Summated limb circumferences | X | X | X | X | X | X | X | X | X | X |
| ED-5D-3L | X | | | | | X | | X | | X |
| LYMQOL | X | | | | X | X | | X | | X |
| Cellulitis recurrence date/s | | | X | | X | X | X | X | X | X |
| Hospitalisation due to cellulitis (date, length of stay) | | | X | | X | X | X | X | X | X |
| Verification of cellulitis recurrence and associated hospitalisation using medical record/general practitioner report | | | X | | X | X | X | X | X | X |
| Intervention provided (type of garment, application of compression bandages) | | X | X | X | X | X | X | X | X | X |
| *Presence of fungal infections/tinea/maceration or cracking of skin between toes* | | | X | | X | X | X | X | X | X |
| Adverse events | | | X | | X | X | X | X | X | X |
| Intervention compliance | | | X | | X | X | X | X | X | X |
| *Occurrence of wounds/ulcers (acute/chronic)* | | | | | X | X | X | X | X | X |

Primary and secondary outcome measures have been underlined. Identified potential risk factors have been italicised.[3,12]
ED-5D-3L, RCT, randomised controlled trial.



BMJ Open: first published as 10.1136/bmjopen-2019-029225 on 15 August 2019. Downloaded from http://bmjopen.bmj.com/ on August 22, 2019 by guest. Protected by copyright.

the final analysis will use a log-rank test with (one-sided) significance level p=0.0238. These efficacy bounds were derived using a Hwang-Shih-DeCani spending function with gamma = −4 to preserve an overall type 1 error rate of 5%.

### Recruitment and enrolment of participants

Recruitment will be conducted over a 2.5-year period. A multifaceted recruitment strategy will be used. In order to capture acute patients (seen in CPHB and Canberra Hospital emergency departments and wards), all patients diagnosed with lower limb cellulitis during their hospital presentation will be sent information regarding the trial and how to contact the CPHB lymphoedema service if they would like to learn more information or self-refer. To recruit from the community, the study will be advertised via posters, radio and articles in various magazines and newspapers, providing information about the trial and encouraging self-referral. Education (in-services, faxes, newsletters and posters) and referral forms will be provided to recruitment sites (Canberra Hospital, CPHB, General Practices within the surrounding region) to encourage health professionals to refer patients. Patients from these sites must consent to a referral to the CPHB lymphoedema service for the study, but do not need to consent to participate in the trial at the time of referral.

After self-referral, a screening phone call will be conducted to check inclusion/exclusion criteria, and for those who appear to be eligible, an appointment at the service will be made with a lymphoedema therapist. At this appointment, candidates will be provided with participant information and consent forms, a verbal explanation of the study and an opportunity to ask questions, prior to choosing to consent or decline to participate.

To promote participation in the study, a free set of compression garments will be offered by a secondary sponsor. Compression garments are expensive, which can provide a barrier to treatment compliance. Participants in the intervention group will receive the free garments at intervention commencement. Participants in the control group will receive the free garments following their first cellulitis recurrence (cross-over) or on study completion for those who do not experience recurrence.

### Patient and public involvement

A patient-centred approach was used to design this study. The trial design replicates the institution's standard clinical practice as closely as possible, while aiming to minimise any additional burden to participants. Patients from the participating clinical service were surveyed to assess acceptability of the model of care undertaken by the trial. As the time required to attend appointments was identified as a potential burden, the trial was designed to minimise scheduled follow-up appointments. Cost of compression therapy was identified as a likely financial burden which is minimised through the provision of two sets of free compression garments and use of accessible funding schemes. Referral processes were developed to enable patients to self-refer to the trial. The cross-over design feature was chosen to ensure participants do not continue to experience episodes of recurrent cellulitis without receiving the institution's standard intervention.

### Assignment of interventions and blinding

Participants will be assigned to the intervention or control group in a 1:1 allocation ratio using block randomisation, with a block size of 10. Sealed sequentially numbered opaque envelopes will be used to ensure concealed allocation. A computer-generated allocation sequence will be created and supplied by a consultant statistician and saved in a folder only accessible by administration staff. Administration staff will prepare the sealed sequentially numbered opaque envelopes, ensuring therapists involved in participant allocation have no premature access to the letters.

Therapists will not be blinded due to the practicalities of providing the intervention within a small team of four specialised clinicians. Further, the visible nature of the treatment and lack of feasible sham interventions prevent effective blinding of both assessors and participants. Additionally, for ethical reasons, participants will be fully informed of both the potential interventions, prior to consenting to participate.

### Data management and quality assurance

Prior to any involvement in the trial, therapists will receive training regarding trial implementation and completion of outcome measures. Refresher training will be provided to therapists annually and the trial protocol will be kept readily available.

For the duration of the study, data will be stored in identifiable form in both a locked office and on a secure access hard drive, accessible only by designated research staff. Data will be entered by a research officer or members of the research team. For quality assurance, data completeness will be reviewed annually, and all entered data will be cross-checked against written records at least once after initial entry. Following trial conclusion and prior to data analysis, all data will be de-identified. Data will be stored for a minimum of 7 years as per CPHB policy, however data may be retained for longer for identified new, ethically approved ancillary studies. A contract with the secondary sponsor ensures that they will have no involvement in the study design, in the collection, analysis, or interpretation of data, in the writing of the manuscript or in the decision to submit the manuscript for publication.

### Participant retention

Once a participant is enrolled in the study, every effort will be made to ensure they are followed up as per the protocol. Where participants cannot attend a scheduled appointment, a phone call assessment may be completed to gain the primary outcome measure. Phone call assessment will not allow for completion of limb volume or QOL measures but will capture the date of cellulitis recurrence and cellulitis-related hospitalisation.

BMJ Open: first published as 10.1136/bmjopen-2019-029225 on 15 August 2019. Downloaded from http://bmjopen.bmj.com/ on August 22, 2019 by guest. Protected by copyright.

Participants can withdraw from the study at any point. For participants who withdraw, the medical record and/or general practitioner report may be checked according to the schedule for cellulitis recurrence and cellulitis-related hospitalisation.

### Termination criteria

Participants will be withdrawn from the study in the case of death, withdrawal of consent or if they develop a wound or lymphorrhoea requiring compression for effective management.[35]

### Proposed methods for data analysis

For the main outcome measure of 'time to the first episode of recurrent cellulitis', survival analysis will be undertaken. Kaplan-Meier plots will be used to visualise patterns of time to first cellulitis recurrence between the groups, with a log-rank test being used to determine if there is a statistically significant difference between the groups. Cox proportional hazards regression may also be used to adjust for important risk factors. Right censoring will be used for participants who are lost to follow-up. Intention to treat analysis will be used, with all enrolled participants being assessed according to their randomisation, regardless of protocol adherence.

For the secondary outcomes of percent change in limb volume and QOL, measures will be taken at multiple time points. Therefore, groups will be compared using a linear-mixed model or using a repeated measures analysis. A generalised linear model will be used to assess the rate of cellulitis-related hospital admissions.

## MINIMISING BIAS

### Selection and attrition bias

Use of randomisation will minimise selection bias and confounding. Stratification will ensure that the use of prophylactic antibiotics is not confounded with treatment assignment. The presence and distribution of other known potential confounding factors will be measured and reported. Intention to treat analysis will be used to prevent attrition bias that may occur through loss to follow-up of participants.

### Internal validity

Use of an RCT and validated measurement tools support the internal validity of this research. The lack of blinding of therapists and participants has the potential to induce surveillance and recall bias and lead to differential measurement error in the reporting of cellulitis recurrence. To minimise this, the accuracy of self-report of recurrence may be cross-checked with the participant's general practitioner or medical record (from CPHB and Canberra Hospital). Diagnosis of cellulitis by doctors external to the study and use of perometry to measure limb volume will reduce the risk of measurement bias and thus differential measurement error. Calibration of the perometer will be performed to prevent non-differential measurement error that could result from machine error.

Control and intervention group participants have the same appointment schedule throughout the duration of the trial, however participants in the intervention group may attend more appointments than the control group. This systematic difference in clinician contact could influence the participant's perceived benefit, allowing potential bias in self-reported measures (LYMQOL, EQ-5D).

Participants enrolled in the trial have a history of two or more episodes of cellulitis diagnosed by medical practitioners independent to the trial. As misdiagnosis of lower limb cellulitis is not uncommon,[36] the trial may include incorrectly diagnosed participants leading to non-differential misclassification.

## ANALYSIS OF COSTS

A within-trial cost-analysis assessment will be conducted. Data obtained from the trial and participant medical records will be used to assess the cost of oedema management and the cost of an episode of cellulitis from both an individual and a health systems perspective. On completion of the RCT, the cost-effectiveness and cost-utility of chronic oedema management to prevent recurrent cellulitis may be assessed.

## ETHICS AND DISSEMINATION

Ethics approval has been granted for these studies by three institutional committees:

1. Calvary Public Hospital Bruce Human Research Ethics Committee (53-2016).
2. Australian Capital Territory Health Human Research Ethics Committee (ETH.4.17.092).
3. University of Canberra Human Research Ethics Committee (cross-institutional approval).

Regardless of the outcome of the trial, the findings are planned to be submitted for publication in relevant peer-reviewed journals and for presentations at national and international conferences. Key findings will be disseminated to identified stakeholders, including primary contact clinicians for patients experiencing cellulitis (doctors and health professionals in acute and community settings), clinicians who manage chronic oedema and professionals who may be involved in developing relevant policy and practice. On request, participants will be provided with a copy of the trial results.

## DISCUSSION

Although current expert consensus recommends compression therapy to prevent the recurrence of cellulitis in patients with lower limb chronic oedema, the evidence supporting this recommendation is lacking. This study aims to review the efficacy of compression therapy to allow for better-informed practice and policy. Given the high incidence of cellulitis within Australia and around the world, reducing cellulitis recurrence will significantly

BMJ Open: first published as 10.1136/bmjopen-2019-029225 on 15 August 2019. Downloaded from http://bmjopen.bmj.com/ on August 22, 2019 by guest. Protected by copyright.

decrease the cost to the healthcare system and reduce the financial and personal burden of sufferers. Further, should compression therapy reduce the recurrence of cellulitis, this may limit the dependence and widespread prescription of prophylactic antibiotics. This trial will be performed on adults receiving healthcare services in the Australian Capital Territory, however, the results will be relevant to cellulitis management throughout Australia and internationally.

**Author affiliations**
[1]Department of Physiotherapy, Calvary Public Hospital Bruce, Canberra, Australian Capital Territory, Australia
[2]Discipline of Physiotherapy, Faculty of Health, University of Canberra, Canberra, Australian Capital Territory, Australia
[3]Biological Data Science Institute, Australian National University, Canberra, Australian Capital Territory, Australia
[4]University of Canberra Research Institute for Sport and Exercise (UC-RISE), University of Canberra, Canberra, Australian Capital Territory, Australia
[5]Calvary Public Hospital Bruce, Canberra, Australian Capital Territory, Australia
[6]Australian National University Medical School, Australian National University, Canberra, Australian Capital Territory, Australia
[7]Australian Institute of Health Innovation, Macquarie University, Sydney, New South Wales, Australia

**Acknowledgements** The authors acknowledge the Calvary Public Hospital Bruce Lymphoedema Team, specifically Gemma Arnold, Marie-Michelle Coulombe, Ingrid Thé, Abby Benton, Emma May, Sarah Squires, Caitlin Norris, Nievelle Chand, Sarah Toohey, Ashlee Cashion and Bhavleen Singh.

**Contributors** EW: trial design and implementation, contribution of original material, editing and approval of final manuscript. BB: trial implementation support and contribution of original material. TN: trial design input and statistical support. VM: economic analysis support. BB, TN, JG, FB and VM: provided supervision, contributed to refinement of the protocol and approved the final manuscript.

**Funding** Calvary Public Hospital Bruce is the primary sponsor, funding clinician time to initiate and manage the trial. Haddenham Healthcare is a secondary sponsor, providing two sets of free compression garments for each trial participant. Haddenham Healthcare had no role in designing this study and will not be involved in the trial implementation, analyses, data interpretation, or publication or dissemination of results. Haddenham Healthcare will not have access to trial data.

**Competing interests** None declared.

**Patient consent for publication** Not required.

**Ethics approval** The Human Research Ethics Committees of Calvary Public Hospital Bruce, Australian Capital Territory Health and University of Canberra all approved this trial.

**Provenance and peer review** Not commissioned; externally peer reviewed.

**Open access** This is an open access article distributed in accordance with the Creative Commons Attribution Non Commercial (CC BY-NC 4.0) license, which permits others to distribute, remix, adapt, build upon this work non-commercially, and license their derivative works on different terms, provided the original work is properly cited, appropriate credit is given, any changes made indicated, and the use is non-commercial. See: http://creativecommons.org/licenses/by-nc/4.0/.

## REFERENCES

1. Swartz MN. Clinical practice. Cellulitis. *N Engl J Med* 2004;350:904–12.
2. Eriksson B, Jorup-Rönström C, Karkkonen K, *et al*. Erysipelas: clinical and bacteriologic spectrum and serological aspects. *Clin Infect Dis* 1996;23:1091–8.
3. Pavlotsky F, Amrani S, Trau H. Recurrent erysipelas: risk factors. *J Dtsch Dermatol Ges* 2004;2:89–95.
4. Inghammar M, Rasmussen M, Linder A. Recurrent erysipelas--risk factors and clinical presentation. *BMC Infect Dis* 2014;14:270–70.
5. The Australian Commission on Safety and Quality in Healthcare. Australian Atlas of Healthcare Variation 2017. 2017:81–90.
6. National Health Performance Authority. Healthy communities: potentially preventable hospitalisations in 2013–14. 2015.
7. National Health Performance Authority. Hospital Performance: costs of acute admitted patients in public hospitals in 2011–12. 2015.
8. Cox NH. Oedema as a risk factor for multiple episodes of cellulitis/erysipelas of the lower leg: a series with community follow-up. *Br J Dermatol* 2006;155:947–50.
9. Jorup-Rönström C, Britton S. Recurrent erysipelas: predisposing factors and costs of prophylaxis. *Infection* 1987;15:105–6.
10. Mortimer PS, Levick JR. Chronic peripheral oedema: the critical role of the lymphatic system. *Clin Med* 2004;4:448–53.
11. Moffatt C, Keeley V, Quere I. The concept of chronic Edema-A neglected public health issue and an international response: the LIMPRINT study. *Lymphat Res Biol* 2019;17:121–6.
12. Dupuy A, Benchikhi H, Roujeau JC, *et al*. Risk factors for erysipelas of the leg (cellulitis): case-control study. *BMJ* 1999;318:1591–4.
13. Karppelin M, Siljander T, Vuopio-Varkila J, *et al*. Factors predisposing to acute and recurrent bacterial non-necrotizing cellulitis in hospitalized patients: a prospective case-control study. *Clin Microbiol Infect* 2010;16:729–34.
14. Chlebicki MP, Oh CC, Cc O. Recurrent cellulitis: risk factors, etiology, pathogenesis and treatment. *Curr Infect Dis Rep* 2014;16:422.
15. Todd M. Chronic oedema: impact and management. *Br J Nurs* 2013;22:623–7.
16. Cox NH, Colver GB, Paterson WD. Management and morbidity of cellulitis of the leg. *J R Soc Med* 1998;91:634–7.
17. Arsenault K, Rielly L, Wise H. Effects of complete decongestive therapy on the incidence rate of hospitalization for the management of recurrent cellulitis in adults with lymphedema. *Rehabil Oncol* 2011;29:14–20.
18. Ko DS, Lerner R, Klose G, *et al*. Effective treatment of lymphedema of the extremities. *Arch Surg* 1998;133:452–8.
19. Thomas KS, Crook AM, Nunn AJ, *et al*. Penicillin to prevent recurrent leg cellulitis. *N Engl J Med* 2013;368:1695–703.
20. Dalal A, Eskin-Schwartz M, Mimouni D, *et al*. Interventions for the prevention of recurrent erysipelas and cellulitis. *Cochrane Database Syst Rev* 2017;6:CD009758.
21. Thomas K, Crook A, Foster K, *et al*. Prophylactic antibiotics for the prevention of cellulitis (erysipelas) of the leg: results of the UK Dermatology clinical trials network's PATCH II trial. *Br J Dermatol* 2012;166:169–78.
22. Sullivan T, de Barra E. Diagnosis and management of cellulitis. *Clin Med* 2018;18:160–3.
23. Raff AB, Kroshinsky D. Cellulitis: a review. *JAMA* 2016;316:325–37.
24. Australasian Lymphology Association. About the National Lymphoedema Practitioners Register. 2018 http://www.lymphoedema.org.au/the-register-updated (Accessed Sep 2018).
25. Carratalà J, Rosón B, Fernández-Sabé N, *et al*. Factors associated with complications and mortality in adult patients hospitalized for infectious cellulitis. *Eur J Clin Microbiol Infect Dis* 2003;22:151–7.
26. Nassaji M, Ghorbani R, Ghashghaee S. Risk factors of acute cellulitis in adult patients: a case-control study. *EJM* 2016;21:26–30.
27. Man IO, Markland KL, Morrissey MC. The validity and reliability of the perometer in evaluating human knee volume. *Clin Physiol Funct Imaging* 2004;24:352–8.
28. Czerniec SA, Ward LC, Lee MJ, *et al*. Segmental measurement of breast cancer-related arm lymphoedema using perometry and bioimpedance spectroscopy. *Support Care Cancer* 2011;19:703–10.
29. Czerniec SA, Ward LC, Refshauge KM, *et al*. Assessment of breast cancer-related arm lymphedema--comparison of physical measurement methods and self-report. *Cancer Invest* 2010;28:54–62.
30. Bulley C, Coutts F, Tan CW. Perometry limb volume measurement: protocol development and reliability. *Eur J Physiother* 2013;15:193–200.
31. Devoogdt N, Lemkens H, Geraerts I, *et al*. A new device to measure upper limb circumferences: validity and reliability. *Int Angiol* 2010;29:401–7.
32. Keeley V, Crooks S, Locke J, *et al*. A quality of life measure for limb lymphoedema (LYMQOL). *J lymphoedema* 2010;5:26–37.
33. Yang Y, Brazier J, Longworth L. EQ-5D in skin conditions: an assessment of validity and responsiveness. *Eur J Health Econ* 2015;16:927–39.
34. R Core Team. R: A language and environment for statistical computing: R Foundation for Statistical Computing, Vienna, Austria. 2018 https://www.R-project.org (Accessed Dec 2018).
35. O'Meara S, Cullum N, Nelson EA, *et al*. Compression for venous leg ulcers. *Cochrane Database Syst Rev* 2012;11:CD000265.
36. Weng QY, Raff AB, Cohen JM, *et al*. Costs and consequences associated with misdiagnosed lower extremity cellulitis. *JAMA Dermatol* 2017;153:141–6.

BMJ Open: first published as 10.1136/bmjopen-2019-029225 on 15 August 2019. Downloaded from http://bmjopen.bmj.com/ on August 22, 2019 by guest. Protected by copyright.

Calvary | Public Hospital Bruce

Patient name:........................................

DOB: ☐☐ ☐☐ ☐☐☐☐
Day Month Year

Study Number: ☐☐ ☐☐

# Core TOOL

**Date completed**: ☐☐ ☐☐ ☐☐☐☐
Day Month Year

**Meets Eligibility Criteria:** Yes ☐ No ☐

| Inclusion Criteria: | Exclusion Criteria |
|---|---|
| • The patients is ≥ 18 years of age<br>Yes ☐ NO ☐<br>• The patient is identified as having chronic oedema (oedema persisting ≥ 3 months) in one or both legs<br>Yes ☐ NO ☐<br>• The patient has a history of ≥ 2 episodes of lower limb cellulitis in the same leg in the 2 years prior to trial referral<br>Yes ☐ NO ☐<br>• The patient is able to understand their involvement in the study, as per the information sheet<br>Yes ☐ NO ☐<br>• The patient is able to give informed consent<br>Yes ☐ NO ☐<br>• The patient has a valid Medicare card<br>Yes ☐ NO ☐<br>• The patient is able to attend regular scheduled appointments<br>Yes ☐ NO ☐ | • The patient is currently receiving chronic oedema management that involves compression<br>Yes ☐ NO ☐<br>• The patient declines to participate or is unable to participate for whatever reason<br>Yes ☐ NO ☐<br>• The patient is receiving end of life care<br>Yes ☐ NO ☐<br>• The patient is medically unstable<br>Yes ☐ NO ☐<br>• Chronic wound/ulcer, or a wound/ulcer requiring specialist treatment or treatment that prevents the use of compression garments<br>Yes ☐ NO ☐<br>• The patient is unable to wear compression (unable to don/doff garments or has a medical condition that contraindicates use of compression) Yes ☐ NO ☐ |

| **Prophylactic antibiotics:** |
|---|
| Yes ☐ No ☐ |

| **Referred by:** |
| --- |
| GP ☐ Calvary Public Hospital ☐ Canberra Hospital ☐ Other ☐ ……………………… |
| **Demographics:** |
| Gender: Male ☐ Female ☐<br>DOB: ☐☐ ☐☐ ☐☐☐☐<br>Day Month Year<br>Weight (kg) __ __ __ . __Height (cm) __ __ __ . __ |

| **Relevant comorbidities:** |
| --- |
| Diabetes mellitus: Yes ☐ No ☐ Heart failure: Yes ☐ No ☐<br>Neurological disorder: Yes ☐ No ☐ Chronic venous insufficiency: Yes ☐ No ☐<br>History of fungal infections/tinea between toes (in past 2 years): Yes ☐ No ☐ |

| **Cellulitis:** |
| --- |
| In the past 2 years, how many episodes of cellulitis have you had in your:<br>Left leg: ☐☐ Right leg: ☐☐<br>In the past 2 years, how many times have you been admitted to hospital (including HITH) with a diagnosis of cellulitis?<br>☐☐ |

| **Classification of Lymphoedema** |
| --- |
| ☐ Primary ☐ Secondary<br>If secondary, swelling related to: ☐ cancer ☐ non-cancer<br>If non-cancer, swelling related to (circle the primary cause and tick other contributing factors):<br>Venous ☐ Immobility ☐ obesity ☐<br>Surgery/Trauma ☐ Post-inflammatory ☐ Other ☐………………………… |

| **Lymphoedema History:** |
|---|
| Estimate duration of Lymphoedema:<br>< 6months ☐ 6 Months to 1 year ☐ 1-2yrs ☐<br>2-5 yrs ☐ 5-10 yrs ☐ >10yrs ☐ |

**Objective leg measures**

**Perometer:**
Volume measured from 53mm to 400mm

Volume of : Right Leg: __ __ __ __ __ ml Left leg: __ __ __ __ __ ml

**Circumferential measures** (taken as per CPHB protocol):

| | **Right leg** | **Left Leg** |
|---|---|---|
| **40cm** | | |
| **30cm** | | |
| **20cm** | | |
| **10cm** | | |
| **Y** | | |
| **A (mid foot)** | | |
| **Total** | | |

Total circumferential measures: Right Leg __ __ __ . __cm Left Leg__ __ __ . __cm

**Chronic oedema present in:** Right leg ☐ Left leg ☐ Both legs ☐

**Randomisation:**

Control ☐ Intervention ☐

Calvary | Public Hospital Bruce

Patient name:........................................

DOB: ☐☐ ☐☐ ☐☐☐☐
Day Month Year

Study Number: ☐☐ ☐☐

## Cellulitis Tool: 6 months review due: ☐☐ ☐☐ ☐☐☐☐

**Tool completed:**

In person: ☐ Via phone call: ☐

**Section 1:**

1. **Date contact made with patient:**

   ☐☐ ☐☐ ☐☐☐☐ or unable to contact patient: ☐

   Day Month Year

2. **Was the medical record/ GP report checked checked?** Yes ☐ No ☐

3. **If yes, what date was the Medical record/ GP report checked:**

   ☐☐ ☐☐ ☐☐☐☐

   Day Month Year

4. **Cellulitis has recurred:**

   Yes ☐ (total number of times: ☐☐) No ☐ Unsure ☐

5. **Date cellulitis recurred (1st recurrence):** NA ☐ **Recurrence resulted from:**

   ☐☐ ☐☐ ☐☐☐☐ ........................................

   Day Month Year (e.g. break in skin, tinea, unknown)

   If >1 occurance, list other dates of recurrence (DD/MM/YYYY):.........................................................................

6. **Cellulitis recurrence was identified by:**

   Self-report ☐ Medical record ☐ GP ☐ NA ☐

7. **Was the patient admitted to hospital (including HITH) as a result of cellulitis recurrence?**

   Yes ☐ No ☐ NA ☐

   **Length of admission**: _ _ days (total of all hospitalisations)

   **Number of days admitted**: With HITH: _ _ days

   As a hospital inpatient (excluding HITH): _ _ days

   If >1 hospitalisation, list other dates of hospitalisation.................................................................

8. **Patient reports (or therapist can see evidence of) presence of fungal infections/tinea/maceration or cracking of skin between toes in the last 6 months:**

   Yes ☐ No ☐

9. **Garment compliance- On average how many days does the patient report they wear their garments each week:** __ days NA ☐

10. **Has the patient reported or therapist noted any adverse effects from treatment:**

Yes ☐ No ☐

11. **Has a wound developed on the legs in the past 6 months?** Yes ☐ No ☐

**If yes:** Acute ☐ Chronic ☐ Cause of wound............................

12. **Objective leg measures (only applicable if patient attends appointment):**

**Perometer:**
Volume measured from 53mm to 400mm

Volume of : Right Leg: __ __ __ __ __ ml Left leg: __ __ __ __ __ ml

**Circumferential measures** (taken as per CPHB protocol):

| | **Right leg** | **Left Leg** |
|---|---|---|
| **40cm** | | |
| **30cm** | | |
| **20cm** | | |
| **10cm** | | |
| **Y** | | |
| **A (mid foot)** | | |
| **Total** | | |

Total circumferential measures: Right Leg __ __ __ . __cm Left Leg__ __ __ . __cm

13. **Weight (kg)** __ __ __ . __ **(only applicable if patient attends appointment)**

14. **Over the last 6 months, what intervention has been provided?**

Education ☐ Compression Garments ☐

Compression Garments provided.......................................................................................................
Compression Bandaging ☐ ( ......... many bandages over ........days) (e.g Mon-Fri = 5 days)

Additional details:

Calvary | Public Hospital Bruce

Patient name:.......................................

DOB: ☐☐ ☐☐ ☐☐☐☐

Date Completed:

☐☐ ☐☐ ☐☐☐☐

Study number: ☐☐☐☐

## Quality of Life Tool: LYMQOL (0 Months)

**Do you have swelling in your lower limb?** ☐ Yes ☐ No If no, tool is complete

| **1. How much does your swollen leg affect the following daily activities** (Check one that best applies) | Not at all (1) | A little (2) | Quite a bit (3) | A lot (4) |
|---|---|---|---|---|
| Your walking | ☐ | ☐ | ☐ | ☐ |
| Your ability to bend, e.g. to tie shoelaces or cut toenails | ☐ | ☐ | ☐ | ☐ |
| Your ability to stand | ☐ | ☐ | ☐ | ☐ |
| Your ability to get up from a chair | ☐ | ☐ | ☐ | ☐ |
| Your occupation | ☐ | ☐ | ☐ | ☐ |
| Your ability to do housework | ☐ | ☐ | ☐ | ☐ |

1

| **2. How much does your swollen leg affect the following questions** (Check one that best applies) | Not at all | A little | Quite a bit | A lot |
|---|---|---|---|---|
| How much does the swelling affect your leisure activities? | ☐ | ☐ | ☐ | ☐ |
| How much do you have to depend on other people? | ☐ | ☐ | ☐ | ☐ |
| How much do you feel it affects your appearance? | ☐ | ☐ | ☐ | ☐ |
| How much difficulty do you have finding clothes to fit? | ☐ | ☐ | ☐ | ☐ |
| How much difficulty do you have finding clothes you would like to wear? | ☐ | ☐ | ☐ | ☐ |
| How much difficulty in finding shoes to fit? | ☐ | ☐ | ☐ | ☐ |
| How much difficulty in finding socks/tights/stockings to fit? | ☐ | ☐ | ☐ | ☐ |
| Does the swelling affect how you feel about yourself? | ☐ | ☐ | ☐ | ☐ |
| Does the swelling affect your relationships with other people? | ☐ | ☐ | ☐ | ☐ |
| Does your lymphedema cause you pain? | ☐ | ☐ | ☐ | ☐ |
| Do you have any numbness in your swollen leg? | ☐ | ☐ | ☐ | ☐ |
| Do you have any 'pins and needles' or tingling in your swollen leg? | ☐ | ☐ | ☐ | ☐ |
| Does your swollen leg feel weak? | ☐ | ☐ | ☐ | ☐ |
| Does your swollen leg feel heavy? | ☐ | ☐ | ☐ | ☐ |

2

3

| **3. In the past week** (Check one that best applies) | Not at all | A little | Quite a bit | A lot |
|---|---|---|---|---|
| Have you had trouble sleeping? | ☐ | ☐ | ☐ | ☐ |
| Have you had difficulty concentrating on things, e.g., reading? | ☐ | ☐ | ☐ | ☐ |
| Have you felt tense? | ☐ | ☐ | ☐ | ☐ |
| Have you felt worried? | ☐ | ☐ | ☐ | ☐ |
| Have you felt irritable? | ☐ | ☐ | ☐ | ☐ |
| Have you felt depressed? | ☐ | ☐ | ☐ | ☐ |

4

**4. Overall how you would rate your quality of life at present?** (Circle one number)

**Poor 0 1 2 3 4 5 6 7 8 9 10 Excellent**

**Health Questionnaire**

**English version for Australia**

*Australia (English) © 1997 EuroQol Group EQ-5D™ is a trade mark of the EuroQol Group*

By placing a tick <u>in one box in each group</u> below, please indicate which statements best describe your own health state today.

**Mobility**

| | | |
|---|---|---|
| I have no problems in walking around | ☐ | PLEASE TICK |
| I have some problems in walking around | ☐ | ONE BOX |
| I am confined to bed | ☐ | |

**Personal Care**

| | | |
|---|---|---|
| I have no problems with personal care | ☐ | PLEASE TICK |
| I have some problems washing or dressing myself | ☐ | ONE BOX |
| I am unable to wash or dress myself | ☐ | |

**Usual Activities** *(e.g. work, study, housework, family or leisure activities)*

| | | |
|---|---|---|
| I have no problems with performing my usual activities | ☐ | PLEASE TICK |
| I have some problems with performing my usual activities | ☐ | ONE BOX |
| I am unable to perform my usual activities | ☐ | |

**Pain / Discomfort**

| | | |
|---|---|---|
| I have no pain or discomfort | ☐ | PLEASE TICK |
| I have moderate pain or discomfort | ☐ | ONE BOX |
| I have extreme pain or discomfort | ☐ | |

**Anxiety / Depression**

| | | |
|---|---|---|
| I am not anxious or depressed | ☐ | PLEASE TICK |
| I am moderately anxious or depressed | ☐ | ONE BOX |
| I am extremely anxious or depressed | ☐ | |



*Australia (English) © 1997 EuroQol Group EQ-5D™ is a trade mark of the EuroQol Group*

To help people say how good or bad a health state is, we have drawn a scale (rather like a thermometer) on which the best state you can imagine is marked 100 and the worst state you can imagine is marked 0.

We would like you to indicate on this scale how good or bad your own health is today, in your opinion. Please do this by drawing a line from the box below to whichever point on the scale indicates how good or bad your health state is today.

**Your own health state today**

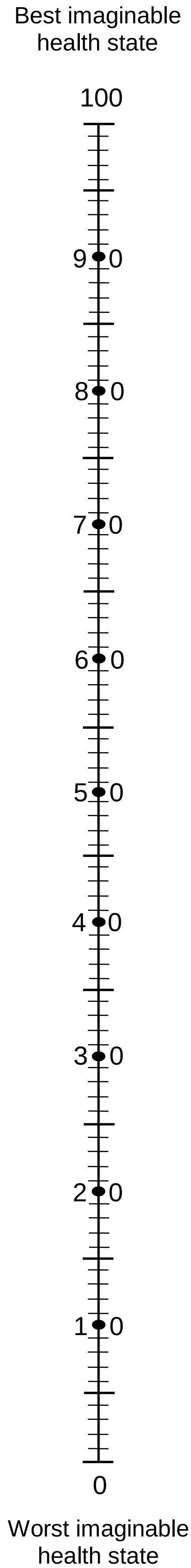




*Australia (English) © 1997 EuroQol Group EQ-5D™ is a trade mark of the EuroQol Group*

Calvary | Public Hospital Bruce

Patient name:........................................

DOB: ☐☐ ☐☐ ☐☐☐☐
Day Month Year

Study Number: ☐☐☐☐

## Infection Tool (used when cellulitis is identified)

| **Tool completed:** |
| --- |
| In person: ☐ Via phone call: ☐ |

| **Section 1:** |
| --- |
| **1. Date contact made with patient:**<br>☐☐ ☐☐ ☐☐☐☐ or unable to contact patient: ☐<br>Day Month Year |
| **2. Was the medical record/ GP report checked checked?** Yes ☐ No ☐<br><br>**3. If yes, what date was the Medical record/ GP report checked:**<br>☐☐ ☐☐ ☐☐☐☐<br>Day Month Year |
| 4. **Cellulitis has recurred:**<br>Yes ☐ No ☐ Unsure ☐ |
| **5. Date cellulitis recurred:**<br>☐☐ ☐☐ ☐☐☐☐<br>Day Month Year<br>**Recurrence resulted from:**<br>........................................<br>(e.g. break in skin, tinea, unknown) |
| **6. Cellulitis recurrence was identified by:**<br>Self-report ☐ Medical record ☐ GP ☐ |
| **7. Was the patient admitted to hospital (including HITH) as a result of cellulitis recurrence?**<br>Yes ☐ No ☐<br>**Length of admission**: _ _ days<br>**Number of days admitted**: With HITH: _ _ days<br>As a hospital inpatient (excluding HITH): _ _ days |
| **8. Patient reports (or therapist can see evidence of) presence of fungal infections/tinea between toes in the last 6 months:**<br>Yes ☐ No ☐ |
| **9. Garment compliance- On average how many days does the patient report they wear their garments each week:** __ days NA ☐ |

**10. Has the patient reported or therapist noted any adverse effects from treatment:**

Yes ☐ No ☐ If yes, please complete required entry in the 'Advserse Events Form'.

**11. Objective leg measures (only applicable if patient attends appointment):**

**Perometer:**
Volume measured from 53mm to 400mm

Volume of : Right Leg: __ __ __ __ __ ml Left leg: __ __ __ __ __ ml

**Circumferential measures** (taken as per CPHB protocol):

| | **Right leg** | **Left Leg** |
|---|---|---|
| **40cm** | | |
| **30cm** | | |
| **20cm** | | |
| **10cm** | | |
| **Y** | | |
| **A (mid foot)** | | |
| **Total** | | |

Total circumferential measures: Right Leg __ __ __ . __cm Left Leg__ __ __ . __cm

**12. Weight (kg)** __ __ __ . __ kg **(only applicable if patient attends appointment)**

**13. Over the last 6 months, what intervention has been provided?**

Education ☐ Compression Garments ☐

Compression Garments provided.......................................................................................................
Compression Bandaging ☐ ( ......... many bandages over ........days (e.g Monday to Friday = 5 days)

Additional details:

Calvary | Public Hospital Bruce

Patient name:........................................

DOB: ☐☐ ☐☐ ☐☐☐☐
Day Month Year

Date of Randomisation: ☐☐ ☐☐ ☐☐☐☐
Day Month Year

Study Number: ☐☐ ☐☐

# Assessment at cross-over (day compression garments are fitted)

**Assessment at cross-over**

1. **Date of cross-over ( date compression garments are fitted)**

☐☐ ☐☐ ☐☐☐☐
Day Month Year

2. **Objective leg measures:**

**Perometer:**
Volume measured from 53mm to 400mm

Volume of : Right Leg: __ __ __ __ __ ml Left leg: __ __ __ __ __ ml

**Circumferential measures** (taken as per CPHB protocol):

| | Right leg | Left Leg |
|---|---|---|
| **40cm** | | |
| **30cm** | | |
| **20cm** | | |
| **10cm** | | |
| **Y** | | |
| **A (mid foot)** | | |
| **Total** | | |

Total circumferential measures: Right Leg __ __ __ . __cm Left Leg__ __ __ . __cm

3. **Weight _ _ _. _ kg**

Calvary | Public Hospital Bruce

Patient name:……………………………………

DOB: | | | | | | | | |
Day Month Year

Date of Randomisation: | | | | | | | | |
Day Month Year

Study Number: | | | | |

## Assessment at cross-over (day compression garments are fitted)

**4. The intervention involved:**

Education ☐

Compression Garments ☐
Compression Garments provided……………………………………………………………………………………………..
Compression Bandaging ☐ ( ……… many bandages over ……..days (e.g Mon-Fri = 5 days)

<u>Additional details:</u>

Calvary | Public Hospital Bruce

Patient name:.......................................

DOB: | | | | | | | |
Day Month Year

Study Number: | | | |

# Exit Form

**1. Date patient exits study**:

| | | | | | | | |
Day Month Year

**2. Primary reason for exiting the study:**

- ☐ Completed study
- ☐ Lost to follow up
- ☐ Death
- ☐ Adverse event
- ☐ Relocation of home
- ☐ Withdrawal by subject (Reason.......................................)
- ☐ Other ...................................................

Additional details:

**Summary of changes to the protocol approved by the ethics committees:**

1. The recruitment strategy was modified

After experiencing very slow recruitment we modified our recruitment strategy in 2017. In addition to providing education to identified healthcare providers and advertising in the community, the ethics committee approved us to mail recruitment letters to all patients who received a diagnosis of lower limb cellulitis within the main public hospitals in Canberra. The recruitment letter provided information regarding the trial and how to contact the lymphoedema service to self-refer or to gain further information.

2. Introduction of an interim analysis and a subsequent increase in the sample size

In 2018, we planned for an interim analysis to be performed by an independent data monitoring committee after the 23$^{rd}$ measured episode of cellulitis. With the addition of a planned interim analysis, the proposed sample size was increased from 152 to 162.

3. Addition of a second quality of life tool (EQ-5D-3L)

In addition to the condition specific QOL tool (LYMQOL), a second generic QOL tool was added to the secondary outcome measures in 2018. The tool was added as it has utility, allowing it to be used for cost-effectiveness analyses.

4. Addition of two surveys to assess the cost of cellulitis and the cost of oedema management

In 2018, two additional participant surveys ('cost of oedema management' and 'cost of cellulitis') were added to the assessment tools to allow us to assess the cost of oedema management and cellulitis from both an individual and a healthcare perspective.

**<u>Original Statistical Analysis Plan</u>**

**Introduction**

This document outlines the planned statistical analysis for the Impact of Compression Therapy on Cellulitis (ICTOC) trial.

**Consent**

All participants will provide written informed consent prior to randomisation.

**Randomisation**

Participants will be assigned to the intervention or control group in a 1:1 allocation ratio using block randomisation, with a block size of 10. Sealed sequentially numbered opaque envelopes will be used to ensure concealed allocation. A computer-generated allocation sequence will be created and supplied by a consultant statistician and saved in a folder only accessible by administration staff. Administration staff will prepare the sealed sequentially numbered opaque envelopes, ensuring therapists involved in participant allocation have no premature access to the letters. Randomisation of participants will be stratified by prophylactic antibiotic use.

**Eligibility Criteria**

*Inclusion criteria:*

- ≥ 18 years of age.
- Two or more episodes of cellulitis diagnosed in the same leg in the past 2 years (at the time of referral).
- Chronic oedema (oedema persisting 3 months) in the leg(s) that have had recurrent cellulitis diagnosed (presence of oedema confirmed by an accredited lymphoedema therapist through interview and physical examination, including a thorough medical history combined with limb palpation and visual assessment).

- Understanding of involvement in the study as per the participant information sheet.
- Provision of informed consent.
- Able to attend regularly scheduled appointments for the duration of the study.
- Has a valid Medicare number.

*Exclusion criteria:*

- Currently wearing effective compression garments (≥compression class 2, or compression class 1 if considered effective by a lymphoedema therapist) regularly (≥5 days per week).
- Declines to participate or is unable to participate for whatever reason.
- Receiving end of life care.
- Medically unstable.
- Chronic wound/ulcer, or a wound/ulcer requiring specialist treatment or treatment that prevents the use of compression garments.
- Unable to wear compression (unable to don/doff garments or has a medical condition that contraindicates the use of compression).

**Research Objectives**

For adults with a history of chronic oedema and recurrent cellulitis who are accessing healthcare services within the ACT, we plan to investigate:

- If use of compression therapy delays the incidence of recurrent cellulitis in the affected limb/s (primary objective),
- If use of compression therapy decreases affected limb volume,
- If use of compression therapy improves quality of life, and
- If use of compression therapy reduces the rate of cellulitis-related hospital presentations

**Research Endpoints**

*Primary endpoint*:

The primary outcome is 'observed time from randomization to the first episode of cellulitis recurrence'. Cellulitis recurrence will only be assessed in a leg that has been assessed as having chronic oedema. If cellulitis occurs in a leg that was not previously identified as having chronic oedema, the infection will not be considered a recurrence.

Cellulitis will be diagnosed by medical practitioners external to the study. Date of cellulitis recurrence (and associated hospitalisation) will be gained by participant self-report and may be verified using medical records from the hospitals and/or general practitioners.

Participants who do not have an episode of cellulitis recurrence during the trial will be censored at their last follow-up appointment.

*Secondary endpoints:*

1. Rate of cellulitis-related hospital presentations:
    a. Cellulitis-related hospitalisations will be measured in the same manner time to cellulitis recurrence
2. Change in leg volume (% and actual) from baseline
    a. Every 6-months from enrolment leg volume will be measured using a perometer between 53 and 400 mm from the ground
    b. Where limb volume cannot be measured using the perometer due to impaired mobility of a participant or equipment failure, summated circumferential leg measurements will be used. Circumferential leg measures will be taken at the mid foot, oblique ankle and at 10, 20, 30 and 40 cm intervals up the leg using a measurement board
    c. Change in leg volume will be calculated based on the volume measurement taken during the baseline measures. For both ipsilateral and bilateral oedema, the same leg (not the contralateral leg) will be used as a comparison.
3. Change in QOL measured using LYMQOL
    a. LYMQOL will be measured at enrolment, 6-months post randomization and then annually post enrolment until the end of the trial.

Where participants cannot attend a follow-up appointment, phone call assessments will be conducted to gain information about cellulitis recurrence and associated hospitalisations. The LYMQOL and EQ-5D may be mailed to the participant to complete.

**Sample Size and Interim Analysis**

Based on the assumptions that compression therapy will reduce incidence of recurrent cellulitis by 50% (Arsenault, Rielly, & Wise, 2011; Ko, Lerner, Klose, & Cosimi, 1998), and that the cellulitis recurrence rate is approximately 40% (Cox, 2006; Jorup-Ronstrom & Britton, 1987), in order to achieve a statistically significant effect (P=0.05) we plan to admit 152 participants (76 per arm) into the study for a follow up of three years or until 45 events of recurrent cellulitis occur. This sample size has been calculated for the primary objective.

**Intention To Treat**

Intention to treat analysis will be used, with all enrolled participants being assessed according to their randomisation, regardless of protocol adherence.

**Baseline Characteristics**

Prior to randomization the following baseline measures are to be collected:

- Age (at date of randomization)
- Gender
- Weight
- Height
- Prophylactic antibiotic use
- Referral source
- Relevant comorbidities (diabetes mellitus, heart failure, neurological disorder, chronic venous insufficiency, tinea pedis),
- Number of episodes of cellulitis in the 2 years prior to referral
- Number of hospital admissions for cellulitis in the 2 years prior to trial referral

- Classification of chronic oedema (primary/secondary)
- Primary cause of chronic oedema and other factors contributing to chronic oedema
- Duration of chronic oedema
- Leg volume (perometer and circumferential measures)
- Leg(s) with oedema

Baseline characteristics will be tabled according to treatment allocation to assess for appropriate balance.

**Treatment Compliance and Adverse Events**

Treatment compliance and adverse effects will be gained by patient self-report at each follow-up appointment.

**Statistical Analysis:**

Data will be entered into excel spreadsheets in a form appropriate for statistical analysis.
Analysis of data will be performed using STATA software

*Primary endpoint:*

For the primary outcome, survival analysis will be undertaken.
Kaplan-Meier plots will be used to visualise patterns of time to first cellulitis recurrence between the groups.
A log-rank test will be used to determine if there is a statistically significant difference between the groups.
The median time to first cellulitis recurrence will be reported for both groups.
Cox proportional hazards regression will also be used to assess the contribution of identified known risk factors (BMI, tinea pedis, wound, history of ≥3 episodes of cellulitis).
Participants who were lost to follow-up will be right censored.

*Secondary endpoints:*

All secondary outcomes will be tested at a significant level of 0.05, and there will be no adjustment for multiplicity.

*Leg volume and QOL:*

For leg volume and QOL measures, groups will be compared using mixed effects linear models or repeated measures analysis.

**Additional analysis**

A secondary per protocol analysis may be performed.

# Final Statistical Analysis Plan

## Introduction

This document outlines the planned statistical analysis for the Impact of Compression Therapy on Cellulitis (ICTOC) trial.

The primary aim of this trial is to determine if compression therapy can prevent recurrence of leg cellulitis in patients with chronic oedema.

This is a single-centre randomised controlled trial with cross-over. Following an episode of cellulitis recurrence, participants in the control group will be crossed over to receive the intervention, whereas intervention group participants will remain in their original group.

Analysis for the primary research paper will only include data from participants whilst they remain in their original assigned group; there will be no analysis of data post cross-over.

Total trial duration is planned for 3.5 years or until 45 episodes of cellulitis to occur. Participants will be followed up for a maximum of 3 years, with the duration of follow-up varying based on time of enrolment.

## Consent

All participants will provide written informed consent prior to randomisation.

## Randomisation

Participants will be assigned to the intervention or control group in a 1:1 allocation ratio using block randomisation, with a block size of 10. Sealed sequentially numbered opaque envelopes will be used to ensure concealed allocation. A computer-generated allocation sequence will be created and supplied by a consultant statistician and saved in a folder only accessible by administration staff. Administration staff will prepare the sealed sequentially numbered opaque envelopes, ensuring therapists involved in participant allocation have no premature

access to the letters. Randomisation of participants will be stratified by prophylactic antibiotic use.

**Eligibility Criteria**

*Inclusion criteria:*

- ≥ 18 years of age.
- Two or more episodes of cellulitis diagnosed in the same leg in the past 2 years (at the time of referral).
- Chronic oedema (oedema persisting 3 months) in the leg(s) that have had recurrent cellulitis diagnosed (presence of oedema confirmed by an accredited lymphoedema therapist through interview and physical examination, including a thorough medical history combined with limb palpation and visual assessment).
- Understanding of involvement in the study as per the participant information sheet.
- Provision of informed consent.
- Able to attend regularly scheduled appointments for the duration of the study.
- Has a valid Medicare number.

*Exclusion criteria:*

- Currently wearing effective compression garments (≥compression class 2, or compression class 1 if considered effective by a lymphoedema therapist) regularly (≥5 days per week).
- Declines to participate or is unable to participate for whatever reason.
- Receiving end of life care.
- Medically unstable.
- Chronic wound/ulcer, or a wound/ulcer requiring specialist treatment or treatment that prevents the use of compression garments.
- Unable to wear compression (unable to don/doff garments or has a medical condition that contraindicates the use of compression).

**Research Objectives**

*Primary objective:*

To determine if compression therapy prevents the recurrence of lower limb cellulitis in adults with lower limb chronic oedema and recurrent cellulitis.

*Secondary Objectives:*

To determine if, in adults with lower limb chronic oedema and recurrent cellulitis, compression therapy:

(1) Prevents cellulitis-related hospital presentations

(2) Reduces affected leg volume

(3) Improves QOL

**Research Endpoints**

*Primary endpoint***:**

The primary outcome is 'observed time from randomization to the first episode of cellulitis recurrence'. Cellulitis recurrence will only be assessed in a leg that has been assessed as having chronic oedema. If cellulitis occurs in a leg that was not previously identified as having chronic oedema, the infection will not be considered a recurrence.

Cellulitis will be diagnosed by medical practitioners external to the study. Date of cellulitis recurrence (and associated hospitalisation) will be gained by participant self-report and may be verified using medical records from the hospitals and/or general practitioners.

Participants who do not have an episode of cellulitis recurrence during the trial will be censored at their last follow-up appointment.

*Secondary endpoints:*

1. Time to cellulitis-related hospital admission
    a. The occurrence of cellulitis-related hospital admissions will be measured in the same manner time to cellulitis recurrence
2. Change in leg volume (% and actual) from baseline

a. Every 6-months from enrolment leg volume will be measured using a perometer between 53 and 400 mm from the ground
b. Fortnightly calibration of the perometer will be conducted using a standardised object of known volume
c. Where limb volume cannot be measured using the perometer due to impaired mobility of a participant or equipment failure, summated circumferential leg measurements will be used. Circumferential leg measures will be taken at the mid foot, oblique ankle and at 10, 20, 30 and 40 cm intervals up the leg using a measurement board
d. Change in leg volume will be calculated based on the volume measurement taken during the baseline measures. For both ipsilateral and bilateral oedema, the same leg (not the contralateral leg) will be used as a comparison.

3. Change in QOL measured using LYMQOL and the EQ-5D-3L.
    a. LYMQOL will be measured at enrolment, 6-months post randomization and then annually post enrolment until the end of the trial.
    b. EQ-5D will be measured at enrolment and then annually until the end of the trial.

Where participants cannot attend a follow-up appointment, phone call assessments will be conducted to gain information about cellulitis recurrence and associated hospitalisations. The LYMQOL and EQ-5D may be mailed to the participant to complete.

**Sample Size and Interim Analysis**

The sample size is calculated for the primary objective.

The sample size assumes the 3-year cellulitis recurrence rate in control participants is approximately 47% and compression therapy will reduce the 3-year incidence of recurrent cellulitis by 50%. Assuming that events occur at a constant rate, these assumptions correspond to a hazard ratio of 0.42.

It is assumed that patients will be recruited over a 2.5-year period, and the total study duration will be 3.5 years. Length of participant follow-up will vary based on time of enrolment. Using a sequential design software package gsDesign in R, in order to detect a hazard ratio of 0.42 with 80% power and 2.5% (1-sided) type 1 error, a total of 45 cellulitis recurrences are needed. Under the present recruitment and recurrence assumptions, we plan to recruit 162 participants (81 per arm).

An interim analysis will be performed by a Data Monitoring Committee after 23 episodes of cellulitis. A log rank test will be used to assess group differences. If a nominal (1 sided) significance level of p=0.003 is detected, indicating a strong clinical effect, the study will be ceased. If the Data Monitoring Committee recommends that the study continue to 45 episodes of cellulitis, the final analysis will use a log rank test with (1-sided) significance level p=0.0238. These efficacy bounds were derived using a Hwang-Shih-DeCani spending function with gamma = -4 to preserve an overall Type I error rate of 5%.

**Intention To Treat**

Intention to treat analysis will be used, with all enrolled participants being assessed according to their randomisation, regardless of protocol adherence.

**Baseline Characteristics**

Prior to randomization the following baseline measures are to be collected:

- Age (at date of randomization)
- Gender
- Weight
- Height
- Prophylactic antibiotic use
- Referral source

- Relevant comorbidities (diabetes mellitus, heart failure, neurological disorder, chronic venous insufficiency, tinea pedis),
- Number of episodes of cellulitis in the 2 years prior to referral
- Number of hospital admissions for cellulitis in the 2 years prior to trial referral
- Classification of chronic oedema (primary/secondary)
- Primary cause of chronic oedema and other factors contributing to chronic oedema
- Duration of chronic oedema
- Leg volume (perometer and circumferential measures)
- Leg(s) with oedema

Baseline characteristics will be tabled according to treatment allocation to assess for appropriate balance.

**Data Quality**

Data completeness will be reviewed annually, and all entered data will be cross-checked against written records at least once after initial entry.

**Treatment Compliance and Adverse Events**

Treatment compliance and adverse effects will be gained by patient self-report at each follow-up appointment.

For treatment compliance, participants will report the number of days per week compression garments were worn in the past 6-months. Treatment compliance rates will be gained by assessing the percentage of participants who wore their garments more then 4 and 5 days per week.

**Statistical Analysis:**

Statistical Analysis was completed using R 3.6.0

*Primary endpoint:*

For the primary outcome, survival analysis will be undertaken.

Kaplan-Meier plots will be used to visualise patterns of time to first cellulitis recurrence between the groups.

A log-rank test will be used to determine if there is a statistically significant difference between the groups.

Cox proportional hazards regression will be used to estimate the hazard ratio which will be presented with reference to the control group.

The median time to first cellulitis recurrence will be reported for both groups.

Number needed to treat will be calculated based on absolute difference in event rate.

Cox proportional hazards regression will also be used to assess the contribution of identified known risk factors (BMI, tinea pedis, wound, history of $\geq 3$ episodes of cellulitis).

Participants who were lost to follow-up will be right censored.

*Secondary endpoints:*

All secondary outcomes will be tested at a significant level of 0.05, and there will be no adjustment for multiplicity.

*Time to cellulitis-related hospital admission:*

Survival analysis will be performed.

Kaplan-Meier plots will be used to visualise patterns of time to first cellulitis-related hospital admission between the groups.

A log-rank test will be used to determine if there is a statistically significant difference between the groups.

Cox proportional hazards regression will be used to estimate the hazard ratio which will be presented with reference to the control group.

*Leg volume and QOL:*

For leg volume and QOL measures, groups will be compared using mixed effects linear models or repeated measures analysis.

**Additional analysis**

A secondary per protocol analysis may be performed.

**Cost-effectiveness:**

A within-trial cost-analysis will be conducted. Data will be obtained from the trial (surveys) and from participant medical records to assess the cost of oedema management and the cost of an episode of cellulitis from both an individual and a health systems perspective.

A secondary paper may present the cost-effectiveness and/or cost-utility of chronic oedema management to prevent recurrent cellulitis.

**Changes in the statistical analysis plan:**

This final statistical analysis plan includes changes made during the study (see protocol changes).

Other changes included:

- The secondary outcome of ‘rate of cellulitis-related hospital admissions’ was changed to ‘time to cellulitis-related hospital admission’; this change was made after determining that the cross-over design prevented an accurate ‘rate’ calculation;
- additional of fortnightly calibration of the perometer;
- defining of how garment compliance is assessed; and
- use R statistical software for data analysis instead of STATA.
